# Supplementary figures and images for: 12 weeks of strength training improves fluid cognition in older adults: A nonrandomized pilot trial
Source: PLoS One. 2021 Jul 22;16(7):e0255018. doi: 10.1371/journal.pone.0255018 (PMC8297768; doi:10.1371/journal.pone.0255018)

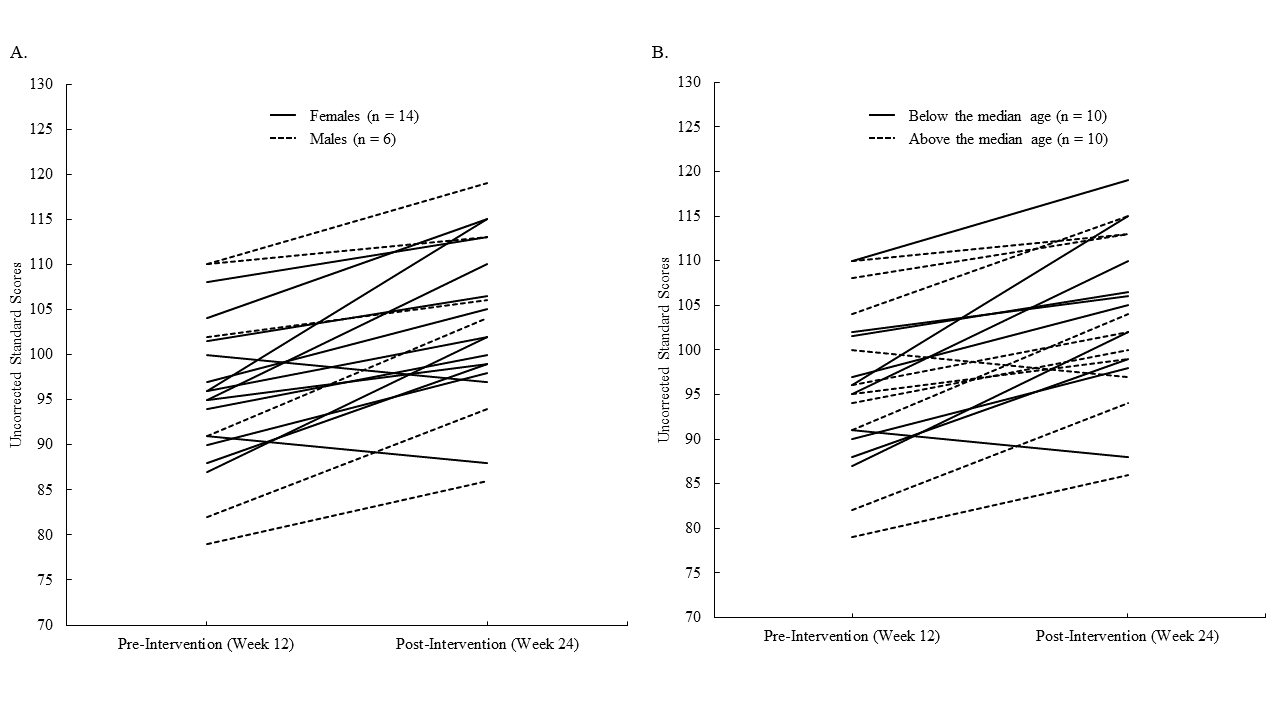

Supplement: S1 Fig — A) Fluid composite scores increased from pre- to post-intervention in both females (+7.4 ± 6.2 Standard Units) and males (+8.5 ± 3.1 Standard Units). B) Using the median age at baseline (68.3 years), we compared the 10 participants below the median age (64.2 ± 2.5 years) and the 10 participants above the median age (74.0 ± 3.7 years). Fluid composite scores increased from pre- to post-intervention in both the participants below the median age (+8.7 ± 5.8 Standard Units) and the participants above the median age (+6.7 ± 4.9 Standard Units). (TIF) [file pone.0255018.s002.tif]
