# Supplementary material for: 12 weeks of strength training improves fluid cognition in older adults: A nonrandomized pilot trial
Source: PLoS One. 2021 Jul 22;16(7):e0255018. doi: 10.1371/journal.pone.0255018 (PMC8297768; doi:10.1371/journal.pone.0255018)
Supplement: S1 Table — (DOCX) [file pone.0255018.s003.docx]

**Table S1** Spearman’s rho correlations between pre- to post-intervention changes in fluid composite score and pre-intervention values of outcome variables of interest (N = 20)

| Measure | Spearman’s rho |
| --- | --- |
| Age | -0.26 |
| IPAQ | -0.20 |
| Body Fat Percentage | -0.03 |
| Lean Body Mass | -0.01 |
| Fat Mass | 0.11 |
| Systolic Blood Pressure | -0.33 |
| Diastolic Blood Pressure | -0.08 |
| Heart Rate | -0.05 |
| Total Body Strength | 0.18 |
| - Lower Body | 0.17 |
| - Upper Body | 0.22 |
| Margaria Power | 0.22 |
| Timed Up and Go | 0.11 |
| Y-Balance^#^ | 0.36 |
| Habitual Gait Speed | 0.14 |
| PSQI Score | -0.22 |
| ISEL Score | 0.15 |

These correlation analyses were used to explore factors that may influence fluid cognitive enhancement after RT. Raw pre- to post-intervention changes in fluid cognition composite score were compared to the single time point of pre-intervention values. For example, the negative spearman’s rho for IPAQ suggests that lower IPAQ responses (lower physical activity levels) at pre-intervention were associated with greater improvements in fluid cognition composite score from pre- to post-intervention.

IPAQ = International Physical Activity Questionnaire.

PSQI = Pittsburgh Sleep Quality Index. Lower scores indicate better sleep quality, scores ≥ 5 are classified as clinically poor sleep quality.

ISEL = International Support Evaluation List. Higher scores indicate greater social support, up to a maximum of 16.

# Four participants were not able to perform all six reach directions at baseline and/or pre-intervention, so their data were excluded from analyses (N = 16).
