# Supplementary material for: 12 weeks of strength training improves fluid cognition in older adults: A nonrandomized pilot trial
Source: PLoS One. 2021 Jul 22;16(7):e0255018. doi: 10.1371/journal.pone.0255018 (PMC8297768; doi:10.1371/journal.pone.0255018)
Supplement: S1 File — (DOCX) [file pone.0255018.s006.docx]

Protocol ID: HS-17-00770

Title: **Cerebrovascular Mechanisms of Cognitive Enhancement after Periodized Resistance Training in Older Adults**

Principal Investigator: Timothy R. Macaulay, CSCS

Division of Biokinesiology and Physical Therapy

University of Southern California

1540 E. Alcazar Street, CHP 155, Los Angeles, CA, 90089

Office: 323-442-2180

Email: [tmacaula@usc.edu](mailto:tmacaula@usc.edu)

Co-Investigator(s): E. Todd Schroeder, PhD

Associate Professor of Clinical Physical Therapy

Director, Clinical Exercise Research Center

Division of Biokinesiology and Physical Therapy

University of Southern California

1540 E. Alcazar Street, CHP 155, Los Angeles, CA, 90089

Email: [eschroed@usc.edu](mailto:eschroed@usc.edu)

Judy Pa, PhD

Assistant Professor of Neurology

Laboratory of Neuro Imaging

Keck School of Medicine of USC

2001 N. Soto Street, SSB1-102, Los Angeles, CA 90032

Email: [jpa@ini.usc.edu](mailto:jpa@ini.usc.edu)

Dominique Duncan, PhD

Assistant Professor of Neurology

Laboratory of Neuro Imaging

Keck School of Medicine of USC

2001 N. Soto Street, SSB1-102, Los Angeles, CA 90032

Email: [dominique.duncan@loni.usc.edu](mailto:dominique.duncan@loni.usc.edu)

Jason Kutch, PhD

Assistant Professor

Director, Applied Mathematical Physiology Laboratory

Division of Biokinesiology and Physical Therapy

University of Southern California

1540 E. Alcazar Street, CHP 155, Los Angeles, CA 90033

Email: [kutch@usc.edu](mailto:kutch@usc.edu)

Lirong Yan, PhD

Assistant Professor

Laboratory of Neuro Imaging

Keck School of Medicine of USC

2001 N. Soto Street, SSB1-102, Los Angeles, CA 90032

Email: [lirong.yan@loni.usc.edu](mailto:lirong.yan@loni.usc.edu)

Biostatistician: Christianne Lane, PhD

Associate Professor

Division of Biostatistics, Department of Preventive Medicine

Keck School of Medicine of USC

2001 N. Soto Street, #202X, Los Angeles, CA 90089

Email: [christianne.Lane@med.usc.edu](mailto:christianne.Lane@med.usc.edu)

Locations: Clinical Exercise Research Center

Division of Biokinesiology and Physical Therapy

University of Southern California

1540 E. Alcazar Street, CHP 149 Los Angeles, CA 90089

The Center for Image Acquisition

Stevens Hall for Neuroimaging

Keck School of Medicine of USC

2025 Zonal Avenue, Los Angeles, CA 90033

**Table of Contents**

[1.0 Background and Hypotheses 5](#_Toc506978218)

[1.1 Disease Background 5](#_Toc506978219)

[1.2 Exercise and the Brain 6](#_Toc506978220)

[1.3 Expertise and Qualifications 8](#_Toc506978221)

[1.4 Hypotheses 9](#_Toc506978222)

[2.0 Objectives and Purpose 9](#_Toc506978223)

[2.1 Primary Objective 9](#_Toc506978224)

[2.2 Secondary Objective 9](#_Toc506978225)

[2.3 Exploratory Objectives 9](#_Toc506978226)

[3.0 Study Design 10](#_Toc506978227)

[4.0 Device Information 11](#_Toc506978228)

[4.1 Magnetic Resonance Imaging (MRI): 11](#_Toc506978229)

[4.2 Dual-energy X-ray absorptiometry (DXA): 11](#_Toc506978230)

[5.0 Selection and Withdrawal of Participants 11](#_Toc506978231)

[5.1 Inclusion Criteria 12](#_Toc506978232)

[5.2 Exclusion Criteria 12](#_Toc506978233)

[5.3 Withdrawal Criteria 13](#_Toc506978234)

[6.0 Randomization Scheme 13](#_Toc506978235)

[7.0 Intervention 13](#_Toc506978236)

[7.1 Periodized RT Intervention 13](#_Toc506978237)

[8.0 Assessment of Efficacy and Safety 14](#_Toc506978238)

[9.0 Clinical and Laboratory Evaluations 14](#_Toc506978239)

[9.1 MRI Scan 15](#_Toc506978240)

[9.2 Cognitive Testing 15](#_Toc506978241)

[9.3 Muscle Strength Testing 15](#_Toc506978242)

[9.4 Physical Function Testing 16](#_Toc506978243)

[9.5 Blood Draw and Analysis 16](#_Toc506978244)

[9.6 Body Composition 17](#_Toc506978245)

[9.7 Blood Pressure 17](#_Toc506978246)

[9.8 Estimated VO2max Testing 17](#_Toc506978247)

[9.9 Questionnaires 17](#_Toc506978248)

[9.10 Study Calendar 19](#_Toc506978249)

[10.0 Criteria for Evaluation and Endpoint Definitions 19](#_Toc506978250)

[11.0 Special Instructions 19](#_Toc506978251)

[12.0 Data Collection and Monitoring 19](#_Toc506978252)

[13.0 Statistical Considerations 19](#_Toc506978253)

[13.1 Statistical Analysis Plan 20](#_Toc506978254)

[13.2 Sample Size and Power Considerations 20](#_Toc506978255)

[14.0 Registration Guidelines 20](#_Toc506978256)

[15.0 Biohazard Containment 21](#_Toc506978257)

[16.0 Ethical and Regulatory Considerations 21](#_Toc506978258)

[16.1 Risk/Benefit Assessment: 21](#_Toc506978259)

[16.2 Financial Obligations and Compensation 21](#_Toc506978260)

[17.0 References 21](#_Toc506978261)

# **1.0 Background and Hypotheses**

## **1.1 Disease Background**

Dementia is an age-related disease caused by neurodegeneration and characterized by progressive cognitive decline that interferes with independent living. The resulting loss of functional independence and reduced quality of life places substantial monetary demands on the individual and the public health system [1]. In the United States alone, the total monetary cost of dementia in 2015 was estimated to be $226 billion [2]. Further calculations suggest that the aging U.S population will result in an 80% increase in total societal costs by 2040 [3]. Worldwide, the prevalence of age-associated cognitive decline and dementia is also steadily increasing. With the rising population of older adults, the number of people with dementia is projected to double every 20 years, reaching 65 million by 2030 and 115 million by 2050 [4].

Although there are many causes of dementia and a wide spectrum of severity, the core clinical criteria for all-cause dementia involves cognitive or behavioral symptoms that interfere with the ability to function at work or at usual activities [5]. These symptoms are generally disclosed to clinicians through complaints from the patient or a knowledgeable informant. Further evidence for longitudinal cognitive decline can be detected though poor performance that exceeds normal age-related declines on objective assessments. Although cognitive symptoms are the hallmark of dementia, behavioral and psychological symptoms such as apathy, depression, and anxiety often dominate both the presentation and course of disease [6]. These symptoms can occur across all stages of dementia with fluctuating prominence. Meanwhile, functional abilities decline in a direct downward trajectory, limiting performance in basic activities of daily living (ADLs) that are essential for independent living. Whether cognitive impairment causes physical impairment, or other dementia-related factors drive functional decline is debatable [7, 8]. Regardless, dementia places considerable strain on patients as well as their families because they require daily caregivers. About 75% of dementia patients are cared for by family or friends at home [9]. In the U.S. in 2014, family members and friends provided 17.9 billion hours of unpaid care to dementia patients, a contribution valued at more than $217 billion [2]. In addition to unpaid hours, caregiver burden has direct impact on the caregivers’ lives, including declines in health and social life [10].

Cognitively normal older adults and patients with mild cognitive impairment (MCI) can exhibit early signs of these symptoms [11], with the exception of functional impairment [12, 13]. Importantly, cognitive and neuropsychiatric alterations are detectable even before the MCI stage [14], highlighting the important distinction between risk factors for dementia and early manifestations of the disease. Indeed, because dementia is not a specific disease but an overall term that describes a wide range of symptoms, there is no clear distinction between its causes and effects. For example, two of the top five most important risk factors for dementia, depression and low cognitive activity, are also symptoms [15]. What is clear is that the risk of dementia increases dramatically once an individual has transitioned to MCI [16]. For example, in the Cache County Study [17] 46% of patients with MCI progressed to dementia within three years, compared to 3.3% of individuals with no history of MCI. Therefore, the most promising strategy to reduce the incidence of disease may be to target modifiable risk factors in seemingly healthy older adults.

With the population of older adults expanding worldwide, and the concomitant rise in cognitively impaired individuals, the above findings highlight the importance of developing interventions to maintain or enhance cognitive function during aging. Current guidelines provide pharmacologic and non-pharmacologic recommendations for symptom management [18, 19]. However, the impact of certain medications on cognition and global functioning is modest and arguably impractical [20, 21]. Furthermore, there are currently no disease-modifying treatments or cures, despite decades of research [22]. Often irreversible brain tissue damage and loss occurs before patients present to clinic with demonstrable cognitive dysfunction [23, 24]. Therefore, successful treatment likely requires presymptomatic neuroprotective therapies [25], as opposed to disease reversal.

Vascular cognitive impairment and dementia (VCID) refers to all forms of cognitive disorder associated with cerebrovascular pathologies. VCID therefore has similar etiologies as stroke, including hypoperfusion, small vessel disease, brain hemorrhage, cerebral infarction, large-artery atherosclerosis, and cardioembolism [26]. Vascular dementia is the second most common form of dementia after Alzheimer’s disease [27], and the prevalence of milder forms of vascular cognitive impairment not qualifying for dementia is even higher [28]. This relationship between vascular disorders and cognitive decline is recognized as a top research priority in all national plans to address dementia [29]. The resulting development of imaging-based biomarkers (such as advanced arterial spin labeling magnetic resonance techniques) and improved understanding of underlying physiological mechanisms has set the stage for promising clinical investigations [30, 31]. The most effective strategy for preventing vascular-related dementia, and to some extent all forms of dementia, may be to target the underlying risk factors for cerebrovascular disease [32, 33].

## **1.2 Exercise and the Brain**

Exercise is a promising strategy to slow or prevent the progression of cognitive decline and dementia [34]. Numerous epidemiological studies indicate that engagement in physical activity improves cognitive function and reduces the risk for dementia. For example, increased physical activity in subjects without neurodegenerative disease predicts a 28% lower risk of developing dementia and a 35-38% reduced risk of cognitive decline [35, 36]. Not only is physical inactivity a top risk factor for dementia [15], but exercise and increased physical activity significantly reduce key cardiovascular risk factors that are associated with dementia [37]. Understanding the neural and physiological processes that underlie these protective effects is essential for the prescription of individually-tailored treatment strategies to promote cognitive health during aging [38].

There are two main exercise modalities: aerobic training (AT) in which large muscles move in a rhythmic manner for sustained periods, and resistance training (RT) which causes muscles to work or hold against an applied force or weight [39]. Most randomized controlled trials (RCTs) investigating effects on the brain have focused on AT. AT has been demonstrated to improve cognitive function and increase brain volume in regions known to be susceptible to age-related brain atrophy [40-42].

As mentioned above, cerebrovascular dysfunction contributes to, and may precede, neuronal dysfunction and cognitive impairment during normal aging and in patients with neurological disorders [43]. An important indicator of preclinical dementia, cerebrovascular function is associated with, and modified by, levels of physical activity [44], making it an important target for therapeutic exercise interventions. Higher levels of self-reported physical activity are associated with improved cerebrovascular structure and function [44, 45]. Cerebrovascular plasticity has been demonstrated after 12 weeks of AT through increased resting cerebral blood flow (CBF) in the hippocampus [46], associated with changes in maximal aerobic capacity, cognitive function, and gray matter volume [47]. Improvements in CBF may therefore predict exercise-induced neuroplasticity and cognitive enhancement. CBF is also negatively associated with markers of endothelial dysfunction in older adults [48], suggesting that improved systemic vascular health may mediate the link between exercise, fitness, and benefits to brain function. This idea is consistent with the theory that vascular senescence may be a common link between declines in cerebral, cardiac, and muscular structure and function with aging [49].

Although RT is recommended for older adults by the American College of Sports Medicine and American Heart Association for its putative role in the protection against sarcopenia [39, 50], its effects on the brain have been less-well studied. Thus, further investigations are needed to understand how RT impacts cerebrovascular health and cognition. Promising cross-sectional evidence supports the efficacy of RT. For example, muscle mass and strength are associated with global cognition [51, 52] and cardiovascular disease risk factors [53, 54], independent of cardiorespiratory fitness. In addition, a recent study by Xu and colleagues [55] indicates that older women who engage in RT at least once per week have greater cerebrovascular perfusion than women who do not. These studies suggest that increased muscle mass, strength, and resting CBF are potential mechanisms by which RT can attenuate or even reverse age-related declines in cognitive function. Exercise intervention studies are necessary to test and extend these observations.

Evidence from RCTs also support the efficacy of RT. Compared to AT, RT has similar, but independent effects on cognition [56-58], cardiovascular risk factors [59], and systemic vascular function [60, 61]. Therefore, RT may impact cognition through vascular mechanisms similar to that of AT. RT may also, however, provide unique benefits to the brain. One meta-analysis found that RT has a larger effect size on executive function (0.53) than AT (0.41), and that this effect size is comparable to that of combined training (0.59) [41]. Unlike AT, RT-induced cognitive enhancement is not associated with changes in aerobic capacity (maximal oxygen uptake), but rather is mediated by increases in muscular strength [62, 63]. These findings support and extend epidemiological literature linking strength with rate of cognitive decline and incident dementia [64]. However, data to support the independent relationship between physical function and cognitive decline are lacking. Further differences between these exercise modalities have been found in imaging studies. While AT-induced improvements in cognitive function are most notably associated with increased hippocampal volume [42, 65], RT-induced improvements are associated with expanded gray matter in the posterior cingulate [66]. RT has also been demonstrated to reverse the progression of white matter hyperintensities [66, 67], an imaging marker of cerebrovascular disease. These studies support the potential for RT to improve cerebrovascular function. However, further investigations are necessary to test this role of RT and determine the mechanisms linking adaptations in strength and cognition after RT.

Another mechanism by which exercise affects the brain is through neurotrophic factors. RT promotes different neurotrophic factors than does AT [68], which may contribute to their distinct physiological pathways of cognitive enhancement. Most studies that investigate the molecular basis of exercise and the brain have been performed after an acute exercise bout. Still, there is ample evidence to suggest that chronic exercise training alters resting serum levels of specific markers. Levels of brain derived neurotrophic factor (BDNF) are lower in frail older adults and patients with neurological disorders than in healthy controls [69, 70]. Both AT and RT have been demonstrated to increase resting BDNF in older adults [71], a putative marker for adult neurogenesis [72]. However, animal studies suggest that AT primarily modulates BDNF/TrkB and beta-CaMKII (calcium/calmodulin-dependent kinase II) pathways, while RT acts on the IGF-1/IGF-1R (insulin-like growth factor/receptor) and AKT pathway [68]. IGF-1 is a primary mediator for the effects of RT on muscle mass, but also passes through the blood brain barrier to stimulate neurogenesis and angiogenesis [73]. Vascular endothelial growth factor (VEGF) also helps regulate basal and exercise-induced angiogenesis [74]. In two human studies, AT did not increase resting BDNF, IGF-1, or VEGF [75, 76]. However, one study found that increased temporal lobe functional connectivity was associated with increased BDNF, IGF-1, and VEGF in the AT group and may be augmented by greater baseline VEGF [75]. The second study found that changes in IGF-1 were positively correlated with changes in hippocampal volume and memory performance [76]. These relationships were independent of fitness, perfusion, or their changes after training, suggesting that changes in IGF-1 levels over time are independently linked to hippocampal volume changes and hippocampus-dependent memory changes. Because RT increases circulating IGF-1 significantly more than AT [77], growth factor response may contribute to the distinct benefits of RT [56]. However, additional research is required to determine the contribution of molecular mechanisms to RT-induced brain adaptations.

## **1.3 Expertise and Qualifications**

Recommendations for strength development include using high loads (≥85% 1RM) for low repetitions (≤6 for core exercises) in two to six sets [78]. In addition, a progressive application of overload is essential to provide the stimulus and recovery necessary to produce optimal results [79]. Recent studies in our lab further demonstrate that short rest interval lengths augment the acute testosterone response to resistance exercise and maximize the gains in muscle size and strength after 12 weeks of periodized strength RT in older adult men [80, 81]. We have established the feasibility of applying a 12 week periodized RT protocol with high compliance rates (complete at least 31 out of 36 sessions plus pre- and post- testing) to healthy older adults (100%) and cancer patients (98.3%) in the Clinical Exercise Research Center (CERC) [80, 82]. We now aim to apply this protocol to a study of brain function in healthy older adults. Magnetic resonance imaging (MRI) and cognitive testing will be performed before and after the intervention, along with previously-used tests of physical function, body composition, and blood draws.

MRI will be performed at The Center for Image Acquisition (CIA) at Stevens Hall for Neuroimaging. The CIA has standard imaging protocols for brain morphometry, resting state brain activity, and cerebral perfusion, including the use of structural MRI, functional MRI (fMRI), and arterial spin labeling (ASL) MRI. Our collaborators at the Laboratory of Neuro Imaging (LONI) have demonstrated the feasibility of conducting an exercise intervention study that includes cognitive testing and MRI in older adults [83]. Furthermore, they have recently developed advanced ASL techniques to non-invasively measure resting CBF, arterial transit time (ATT), and intracranial vascular compliance (VC) [84, 85]. ATT, the duration for blood to travel from large arteries to capillaries, is estimated from multi-delay pseudocontinuous ASL [86]. Obesity (body fat percentage and body mass index) and poor cognitive performance have been found to be inversely related to ATT in patients with coronary artery disease [87]. In addition, by synchronizing ASL with the peak systolic and early diastolic phases of the cardiac cycle, vascular compliance can be calculated as the ratio of the change in cerebral blood volume (CBV) between systole and diastole to the change in arterial blood pressure between systole and diastole [84]. Using this technique, older adults had significantly lower intracranial VC (due to both reduced change in CBV and increased pulse pressure) than young adults. In addition, VC was strongly associated with age, CBF, and aortic stiffness.

The promise of an exercise intervention extends beyond the target disease, having further benefits to the well-being of patients. These types of treatments positively impact fundamental processes of aging, thus reducing the rate of all-cause mortality. Compared to pharmacological treatments, exercise is available at low cost and minimal risk of inducing adverse side effects. Even with moderate benefits to a specific disease like dementia, the global impact on healthcare would be substantial.

## **1.4 Hypotheses**

1. 12 weeks of periodized RT with short rest intervals improves cerebrovascular function (resting CBF, ATT, and intracranial VC) in healthy older adults 60-80 years of age.
2. A 12-week periodized RT program designed specifically to maximize strength gains improves cognition in healthy older adults.

# **2.0 Objectives and Purpose**

We propose a mechanistic proof-of-concept intervention to investigate the effects of periodized resistance training (RT) on brain health and the underlying vascular processes that mediate cognitive enhancement in older adults. The overall goal of this proposal is to apply a 12-week periodized RT program that we have previously demonstrated to optimize strength gains in older adults to a collaboration with the Laboratory of Neuro Imaging (LONI) at the University of Southern California to determine the effects on brain health in older adults. Arterial spin labelling (ASL) magnetic resonance imaging (MRI) and the NIH Toolbox® Cognition Battery will be used to assess cerebrovascular function and cognition before and after a 12-week periodized RT program in 23 healthy older adults 60-80 years of age. Participants will serve as their own controls in an initial 12-week control period, and henceforth will all receive 12 weeks of supervised RT.

To address this goal, we propose the following aims:

## **2.1 Primary Objective**

To determine whether 12 weeks of periodized RT improves cerebrovascular function in healthy older adults. Cerebrovascular function will be evaluated by resting CBF, intracranial vascular compliance, and arterial transit time (ATT), assessed using ASL MRI techniques. This study will establish response variance and effect sizes while generating preliminary signals of efficacy in an aging related outcome.

## **2.2 Secondary Objective**

To examine the effects of periodized RT on cognition in healthy older adults. The NIH Toolbox® Cognition Battery will be administered to assess executive function, attention, episodic memory, language, processing speed, and working memory. In addition to the individual measure scores, this battery will yield the following summary scores: cognitive function composite score, fluid cognition composite score, and crystalized cognition composite score.

## **2.3 Exploratory Objectives**

1. To determine whether changes in cerebrovascular function mediate the relationship between RT and improvements in cognition.

2. To evaluate intervention effectiveness and explore potential mechanisms of benefit using various physical, physiological, and biochemical outcomes. These outcome measures include: 1) physical capacity (muscle strength and maximal oxygen consumption), body composition, and functional mobility; 2) putative blood markers of exercise-induced brain plasticity (i.e. BDNF, IGF-1, and VEGF); 3) cardiovascular risk factors (i.e. blood pressure) and markers of endothelial dysfunction (i.e. tissue plasminogen activator (t-PA) and Von Willebrand factor (VWF)); and 4) brain morphology (e.g. volume, cortical thickness, and white matter lesions) and resting state brain activity.

3. To investigate associations between brain adaptations and potential mechanisms of benefit. Changes in the above outcome measures will be examined for their roles in mediating RT-induced changes in cognition and cerebrovascular function.

# **3.0 Study Design**

This study will be conducted in the Clinical Exercise Research Center (CERC) in the Division of Biokinesiology and Physical Therapy on the University of Southern California Health Sciences Campus. In this mechanistic proof-of-concept clinical trial, the effects of a 12-week periodized RT intervention on the brain will be compared to that of an initial control period of equal duration. After baseline testing, participants will be asked to resume normal life for the 12-week control period. After the control period, and before the RT intervention, participants will undergo the same tests performed at baseline. Post-intervention, participants will be tested again (please see study calendar 9.10).

**Study Visit 1:** Interested participants will report to the CERC and read the informed consent with a study investigator. The prospective participant will be given the opportunity to ask any questions they may have about the study. If the potential subject is willing to take part in the study, she/he will sign the informed consent agreeing to participate. She/He will also complete a Physical Activity Readiness Questionnaire (PAR-Q) to ensure she/he is at a baseline fitness level necessary for exercise training, and a Mini-Mental State Examination (MMSE) to screen for cognitive deficits. All potential subjects will have their blood pressure taken during this visit to ensure normal levels. Additional screening will take place, according to the inclusion/exclusion criteria.

**Study Visit 2 (Baseline Testing Part 1):** All participants will undergo the following tests: cognitive testing, quality of life questionnaires, and MRI scans. Approximate time: 2.5 hours

**Study Visit 3 (Baseline Familiarization Day):** Descriptive factors will be measured on all participants. Then they will undergo exercise and testing familiarization. Approximate time: 1.5 hours

**Study Visit 4 (Baseline Testing Part 2):** All participants will undergo the following tests: International Physical Activity Questionnaire (IPAQ), blood pressure, body composition, blood draw, Physical Function tests, Muscle Strength tests, and estimated VO2max tests. Approximate time: 2.5 hours

**Control Period:** Participants will be encouraged to continue normal activities of daily living and eating routines.

**Study Visit 5 (Pre-Intervention Testing Part 1):** All participants will undergo the following tests: cognitive testing, quality of life questionnaires, and MRI scans. Approximate time: 2.5 hours

**Study Visit 6 (Pre-Intervention Testing Part 2):** All participants will undergo the following tests: International Physical Activity Questionnaire (IPAQ), blood pressure, body composition, blood draw, Physical Function tests, Muscle Strength tests, and estimated VO2max tests. Approximate time: 2.5 hours

**Intervention Period:** The RT intervention will consist of 36 sessions. Participants will perform all sessions at the CERC (3 days per week for 12 weeks), supervised by an exercise specialist. A two-week buffer period will be allowed for participants to make-up missed sessions (up to 6 session, 3 per week).

**Study Visit 7 (Post-Intervention Testing Part 1):** All participants will undergo the following tests: cognitive testing, quality of life questionnaires, and MRI scans. Approximate time: 2.5 hours

**Study Visit 8 (Post-Intervention Testing Part 2):** All participants will undergo the following tests: International Physical Activity Questionnaire (IPAQ), blood pressure, body composition, blood draw, Physical Function tests, Muscle Strength tests, and estimated VO2max tests. Approximate time: 2.5 hours

# **4.0 Device Information**

## **4.1 Magnetic Resonance Imaging (MRI):**

A 3T MRI scanner (Siemens, Malvern, PA) will be used to measure brain morphology (e.g. volume, cortical thickness, hippocampal subfields, and white matter structure and lesions), resting state brain activity, and cerebrovascular function (arterial spin labeling, ASL). Participants will be positioned supine in the MRI scanner with head and neck support and earplugs for approximately 1 hour. An MRI scan is a non-invasive, painless radiology technique that has the advantage of avoiding x-ray radiation exposure. There are no known side effects of an MRI scan. All scanning sessions will have a minimum of two trained personnel with their credentials approved by the Center for Image Acquisition (CIA). MRI safety policies and procedures are described on the CIA website: <http://cia.ini.usc.edu/home/policy_safety>.

## **4.2 Dual-energy X-ray absorptiometry (DXA):**

DXA scans (Lunar iDXA, GE Healthcare, Waukesha, WI) are used in body composition analysis to measure three body components: bone mineral mass, fat mass, and lean soft tissue mass. A dual-energy x-ray source is required to determine the areal densities of up to two components of an absorber. By considering the body to be made up of a series of two component systems (e.g. fat mass and fat free mass; soft tissue and bone mineral; fat and lean soft tissue), DXA analyzes each system separately and then combines the results for a complete body composition analysis. The mass fraction of each component is determined using the R-value (ratio of low energy mass attenuation coefficient to the high energy mass attenuation coefficient) and mass attenuation coefficients of each component.

Participants will be positioned supine on the scanner for approximately 12 minutes to complete a whole body, spine, and hip scan. Although the DXA is an x-ray device, the radiation dose is so low that the risk to patients is unknown.

# **5.0 Selection and Withdrawal of Participants**

Method of subject identification and recruitment: 23 healthy older adults, age 60-80yrs, will be recruited from the local communities surrounding the USC Health Sciences Campus. They will be assigned a subject identification number (PID) after signing of the informed consent. Recruitment strategies, as well as plans for recruitment to replace potential dropouts during the course of the study, will include flyer distribution (e.g., around the Health Sciences Campus) and, whenever possible, discussion of the study protocol via personal solicitation. The USC Health Sciences Campus is approximately 5 miles from residential areas/business areas, which provide access to health clubs, country clubs, and local gyms, allowing for recruitment of the required number of participants. Only investigators and staff will approach the participants, introduce themselves (stating their name and association with USC and the Clinical Exercise Research Center), discuss the study briefly with the participant (including the time requirements, training protocols, and blood draws), inquire about possible interest on the part of the participant, and, if the participant is interested, obtain a contact number for follow-up. Patients who are not competent to give informed consent will not be recruited.

Potential participants will be given an opportunity to ask questions and seek additional information about the study and alternatives. They will be encouraged to take the informed consent home to read over and discuss with relatives, friends, physician, etc. If they subsequently desire additional information about the study, they will be invited to return for the opportunity to ask more questions. Participants will not be included in the study unless the informed consent is obtained from the participants themselves. The participant will be asked if she/he understands the information in the informed consent and relate that back to the investigator(s).

Documentation of consent: Timothy R. Macaulay, CSCS

E. Todd Schroeder, PhD

Privileges/certificates and licenses: Dr. Todd Schroeder is an Associate Professor in the Division of Biokinesiology & Physical Therapy with a joint appointment in the Department of Medicine. Dr. Schroeder is an Exercise Physiologist, instructor of Clinical Exercise Physiology in the Division of Biokinesiology and Physical Therapy, and director of the Clinical Exercise Research Center (CHP 149). Timothy Macaulay is a doctoral student in the Division of Biokinesiology and Physical Therapy.

Eligibility waivers are not permitted. Patients must meet all of the inclusion and exclusion criteria to be registered to the study. Study treatment may not begin until a subject is registered.

## **5.1 Inclusion Criteria**

The patient must give written informed consent to enter into the study after receiving a full explanation of the study's purpose, procedures, and risks. A copy of the consent form for this protocol will be given to each subject entering the study. All of the following criteria must be met for the subject to be eligible for the study:

- 60-80 year old men and women.
- Community-dwelling and living independently (without need of assistance).
- Interest and availability for participation in a 12-week planned RT program at the CERC, including pre- and post- testing.
- Competency in English sufficient for assessment and training.
- Able to see and hear sufficiently to participate in RT.
- Not engaged in any structured exercise training outside of this intervention.
- Eligible to undergo MRI.
- Answer NO to all questions on the Physical Activity Readiness Questionnaire (PAR-Q) or receive medical clearance from a physician.

## **5.2 Exclusion Criteria**

Patients that meet any of the following criteria are excluded from entry into the study:

- Possible Dementia (score less than or equal to 23 on MMSE).
- History of known neurological disease (e.g. Epilepsy, Multiple sclerosis, Parkinson disease, Alzheimer’s disease), cerebral infarct (e.g. Stroke), or traumatic brain injury.
- History of known cardiovascular or metabolic disease or chronic illness which may compromise the patient’s ability to safely perform the RT program (e.g. coronary artery disease, arrhythmia, asthma requiring an inhaler during exercise), or presently uncontrolled hypertension (SBP > 140 mmHg or DBP > 90 mmHg). Patient may be required to provide clearance from a physician at the study team’s discretion.
- Type 1 or Type 2 Diabetes.
- Changes in chronic pharmacological treatment (e.g. Aspirin, Statins, or ACE inhibitors) or hormone therapy during the intervention period.
- Current treatment for congestive heart failure, angina, uncontrolled arrhythmia, DVT or other cardiovascular event.
- Myocardial infarction, coronary artery bypass grafting, angioplasty or other cardiac condition in the past year.
- Contraindications against MRI (e.g. metal implants, claustrophobia).
- Self-report regular heavy RT in the past 6 months (i.e. “strengthening exercises or lifting weights heavy enough that you could not perform more than 15 repetitions in one set”).
- Musculoskeletal injuries interfering with the ability to perform RT or medical conditions for which exercise in contraindicated.

## **5.3 Withdrawal Criteria**

Patients may be withdrawn from the study due to injury, illness, personal choice, or non-compliance that would prevent participants from completing all visits.

# **6.0 Randomization Scheme**

No randomization will be necessary. Participants will serve as their own controls, and therefore will all receive supervised RT. The 12-week control period will be done before the RT intervention to ensure that results are not confounded by a detraining effect. In addition, long-term cognitive benefits after exercise interventions have been found up to two years follow-up [88, 89].

# **7.0 Intervention**

Participants will be encouraged to continue normal activities of daily living and eating routines outside the resistance training protocol of the present study. Participants may be contacted for scheduling, but will not be monitored during the control period. All participants will be asked to abstain from strenuous activity 24 hours prior to ALL study visits. During the training workouts and testing days, participants will be allowed to drink water ad libitum.

## **7.1 Periodized RT Intervention**

A periodized and progressive total-body resistance training program emphasizing development of lower and upper body strength will be performed 3 days/week for 12 weeks. Each training session will be supervised at the CERC, will be approximately 1 hour in duration, and will begin with a 5-minute warm-up of light bodyweight resistance exercises and stretching exercises (if needed). Throughout the training program, sets/exercise will range from 3-5 and repetitions from 6-4. The rest interval between sets will be 60 seconds for all exercises. Training logbooks will be kept for each participant and will detail training loads, sets, and repetitions for each exercise for every training session. All training loads, sets, and repetitions performed by the participants will be recorded by study investigators.

Exercise order will be controlled by the investigators during all training sessions. Throughout the entire 12-week resistance training program, all sets will be performed maximally for the assigned number of repetitions. The training loads used will be individually progressed in a safe and effective manner, in order to employ a progressive overload/challenge to the neuromuscular system and elicit the greatest training-induced neuromuscular adaptations possible. Static stretching exercises will be performed at the end of every RT session.

Sample Weekly RT Intervention Protocol

| Day 1 | Day 2 | Day 3 | Day 4 | Day 5 | Day 6 | Day 7 |
| --- | --- | --- | --- | --- | --- | --- |
| RT (CERC) | Rest | RT (CERC) | Rest | RT (CERC) | Rest | Rest |

# **8.0 Assessment of Efficacy and Safety**

Side-effects will be monitored. Participants will be asked about the following side-effects at the beginning of each testing and training session:

- energy level
- muscle fatigue and soreness
- joint stiffness
- pulls/strains
- sleep quality

Other side-effects will be documented if presented by the participant: everyday strength, everyday energy levels, appetite, thirst, sleep quality, or participation in strenuous activity or exercise within 24 hours prior to the visit.

Adverse events will be documented by the PI and the IRB will be notified of any severe adverse events (SAEs) within 24 hours of study team becoming aware of such SAE event. Adverse events (AE) that may be associated with this study include injury or excessive soreness from testing or the resistance training program. No serious adverse events (SAEs) are expected with this study.

No long-term toxicities are to be monitored after completion of the study. No long-term adverse side effects have been associated with resistance training.

# **9.0 Clinical and Laboratory Evaluations**

Assessments performed exclusively to determine eligibility for this study will be done only after obtaining informed consent. All screening procedures must be performed within 30 days prior to registration unless otherwise stated.

Descriptive factors to be collected include age, height, weight, gender, chronic pharmacological treatment (e.g. ACE inhibitors, Statins, Aspirin), treated pathologies (diabetes, hypertension, high cholesterol, anemia), and hormone therapy status.

Measurements will be conducted over two study visits at each of the following time points for all study outcomes:

1. Weeks 1-2 (Baseline)

2. Week 15 (Pre-intervention)

3. Weeks 28-29, 29-30, or 30-31 (Post-intervention)

Each of these 6 study visits are anticipated to take approximately 2.5 hours. In addition, one familiarization session (~1.5 hours) will be performed during baseline testing.

Buffer Period: In the event a subject in the RT group misses a training session during the 12-week intervention (i.e. has not completed all 36 training sessions by the end of study week 15), she/he will be allowed to complete missed sessions during a 2-week buffer period following the 12-week intervention. A maximum of 6 sessions (3 per week) will be allowed during this period.

All baseline study procedures will be conducted within 2 weeks prior to the start of the intervention, and all post-test study procedures will be conducted within 2 weeks after the end of the intervention (Section 9.10, Study Calendar).

## **9.1 MRI Scan**

MRI is not invasive, and it does not expose participants to treatments with energy such as X-rays or CT scans do. Many people have been safely studied using MRI techniques. While there are no significant risks from MRI as it is to be performed, the MRI procedures are not appropriate for people with pacemakers or metal in their bodies. Prospective participants will be excluded from this study if they have a pacemaker or any metal in their body that cannot be easily removed. The space inside the bore of the MRI machine is just large enough for an average adult. Because the space is so confined, some people feel claustrophobic once inside the MRI machine. If prospective participants have a history of claustrophobia, or experience it during MRI procedures, they will be excluded from the study. Because the MRI scan makes loud noises, we will give participants ear plugs to dampen the sound. They may also experience peripheral stimulation, which will feel like a gentle tap or sensation of mild muscle tremor. If they do not like being in the scanner for any reason, we will immediately stop the experiment.

Participants will be positioned supine in the MRI scanner with foam head and neck support and earplugs for approximately 1 hour. The three noninvasive MRI techniques that will be used in this study are structural MRI, resting state functional MRI (fMRI) [90, 91], and arterial spin labeling (ASL) MRI [92, 93]. Furthermore, a dynamic ASL technique will be used to assess intracranial vascular compliance by synchronizing ASL with the peak systolic and early diastolic phases of the cardiac cycle to measure arterial cerebral blood volume (CBV) at systole and diastole [84]. Vascular compliance will be calculated as the ratio of the change in CBV between systole and diastole to the change in arterial blood pressure between systole and diastole. An MRI compatible cuff sphygmomanometer will be used to measure brachial blood pressure before and after the MRI scans. MRI compatible ECG leads will be placed on the subject’s chest to trigger vascular compliance scans.

## **9.2 Cognitive Testing**

Participants will be asked to perform various tasks to assess cognitive function. They will be presented with images, words, numbers, and/or sounds on an iPad.

The NIH Toolbox Cognition Battery (NIHTB-CB) and Cognition Supplemental Measures will be administered by a trained study investigator. The battery has been normed and validated across the lifespan in participants ages 3-85 and its use ensures that assessment methods and results can be used for comparisons across existing and future studies [94, 95]. The constructs and cognitive subdomains to be tested include executive function, working memory, episodic memory, language, processing speed, attention, and learning. In addition to individual measure scores, the battery will yield the following summary scores: Cognitive Function Composite Score, Fluid Cognition Composite Score, and Crystallized Cognition Composite Score [96]. The NIHTB-CB was designed to not exceed 30 minutes in duration. From experience, we anticipate the entire cognitive testing session will last approximately 45 minutes in duration, including setup, the battery, and supplemental measures. Three test versions will be used to reduce practice effects [97].

## **9.3 Muscle Strength Testing**

Previous research in older, untrained women suggests that at least one familiarization session is ideal before strength testing is performed [98]. Therefore, one familiarization session will be performed approximately one week prior to strength testing. The purpose of the familiarization training is to increase the reliability of strength measurements during the testing sessions across participants of various training statuses. Participants will be instructed in proper technique, breathing and full range of motion on all resistance exercises that will be used during testing. The exercises performed will include the leg press, leg extension, leg curl, chest press, lat pulldown, seated row and smith machine seated shoulder press. For muscle strength testing, the 7 exercises practiced during the familiarization sessions will be assessed at baseline, pre-intervention, and post-intervention using 4-6 repetition maximum (RM) strength tests. Participants will complete a warm-up of 5-10 repetitions at an approximate 20RM load, which is estimated based on researcher experience and verbal questions pertaining to training history. One additional warmup set may be used if necessary. Participants will rest for one minute before the first RM attempt. Three attempts will be allowed to reach the final 4-6RM load with a 2-minute rest period between attempts.

The load and number of completed repetitions will be used to estimate 1RM according to the equation [99]: Predicted 1RM (lbs) = (weight lifted in lbs)/(1.0278 – (.0278 x reps))

This equation was chosen based on strong correlations between actual and predicted 1RM values in older adults [100]. The 1RM represents a valid assessment of muscle strength in training-specific exercises in young and older adults [101].

## **9.4 Physical Function Testing**

Physical function tests include the Gait Speed Test [102], Margaria Stair Climb [103], Timed Up-and-Go [104], and Y Balance test [105], which will be assessed at baseline and in the post-intervention period.

Gait Speed Test: Gait speed is an important marker of vitality that can predict the life expectancy of older adults [102]. Tests of gait speed have high validity and reliability in both healthy older adults and patients with dementia [106-108]. Participants will be instructed to walk on a marked 8-meter walkway at a normal, comfortable speed. An examiner will walk behind and to the side of the participant to ensure safety without influencing their gait speed. Time taken to walk the central 4 meters of the course (marked by tape) will be recorded to the nearest hundredth of a second to calculate gait speed in meters/second. An average gait speed of 2 trials will be calculated.

Margaria Stair Climb: Functional power will be measured using a stair climb test that has been successfully performed and correlated with lower-extremity power and mobility performance in older adults with a reliability coefficient of 0.99 [109]. Participants will be instructed to ascend a flight of 10 stairs one step at a time as quickly as possible without using a handrail. Timing will begin when one foot steps on the 3rd stair, and ends when one foot reaches the 9th stair. Time is recorded to the nearest .01 second, and an average of 3 trials is calculated. One practice trial will be given before the 3 trials.

Timed Up-and-Go Test: Mobility will be assessed using the Timed Up and Go (TUG) test, which has been shown to predict immediate fall risk better than static balance tests or isometric muscle strength [110]. A reliability coefficient of 0.98 has been reported [111]. Participants begin seated in a chair with hands on the armrests, are asked to rise, walk to a line on the floor 3 m from the chair, turn around, and return to the same seated position as quickly and safe as possible. Scores will be taken as the time to complete the task, with one practice trial given. An average of time for 3 trials will be calculated.

Y Balance Test: Participants will perform the Y balance test (Functional Movement Systems, Danville, VA), a dynamic balance assessment with validity for predicting risk of lower extremity injury, identifying balance deficits, and responding to training programs [112]. Participants will stand on one foot on a stance platform from which 3 pieces of polyvinylcholoride pipe, marked in 5 mm increments, extend in the anterior, posteromedial, and posterolateral directions. The participant will be instructed to push a target with the foot of the reaching limb in each of the 3 directions. Maximal reach is recorded for each foot, in each of the 3 directions. A practice trial is given for each foot in each direction.

## **9.5 Blood Draw and Analysis**

Fasting blood draws will be performed at baseline and post-intervention. Blood samples will be obtained from the participants specifically for this research. All blood samples will be obtained in the Clinical Exercise Research Center. Each blood draw will require two tubes (approximately 20 mL) of blood, and will be performed with new/clean needle sticks and vacutainer tubes. These blood draws will be conducted by licensed personnel, in a controlled environment according to protocols designed for maximum safety. The blood samples will be handled using universal precautions defined by the Centers for Disease Control and Prevention.

At the beginning of testing, the study participants will rest for approximately 15 minutes in the seated position, and then blood samples will be drawn. All blood samples (taken at baseline and post-intervention) will be drawn with the participant in the seated position. To avoid the effects of the circadian rhythm on hormonal concentrations, each study participant will have their blood drawn and perform Testing Part 2 at the same time of day pre- and post-intervention.

BDNF, IGF-1, VEGF, t-PA, and VWF will be assayed in the venipuncture blood. No other laboratory testing will be required for other analytes. The CERC, directed by E. Todd Schroeder, Ph.D., will fund processing and analyses of the assays. These assays are being performed because our study is examining the effects of RT on brain health and vascular function. BDNF, IGF-1, and VEGF are putative blood markers of exercise-induced brain plasticity, and t-PA and VWF are markers of endothelial dysfunction associated with resting CBF.

## **9.6 Body Composition**

Lean body mass (LBM), fat mass (FM), percent body fat (PBF), bone mineral content (BMC), and bone mineral density (BMD) will be measured by dual energy x-ray absorptiometry (DXA). Patients will be positioned supine on the scanner for approximately 12 minutes to complete a whole body, spine, and hip scan. We will also measure total body fat using the InBody 770 (Biospace, Inc) bioelectrical impedance device to compare bioelectrical impedance measures of body fat to DXA measures of body fat in this population. The patients will stand on a scale-like device for approximately 30 seconds while a non-detectable electrical current is passed through their body from the hand and foot contacts to determine impedance from which body fat is calculated.

## **9.7 Blood Pressure**

Blood pressure will be assessed using a sphygmomanometer at the brachial artery while the patient is seated.

## **9.8 Estimated VO2max Testing**

Participants will complete a submaximal treadmill exercise protocol to estimate maximal oxygen consumption (VO2max), a measure of aerobic capacity. The single-stage submaximal treadmill walking test was validated by correlating the estimated VO2max to the measured VO2max (R^2^ = 0.92) [113]. A polar heart rate monitor will be used to measure heart rate during the test. Participants will warm-up for 4 minutes at a 0% grade and a walking speed (recommended speed: 3.4-4 mph) that brings their heart rate to 50%-70% of her/his age-predicted max heart rate (220-age). After the warmup, the treadmill grade will be increased to 5%, and the participant will continue to walk at the same speed for an additional 4 minutes. Steady-state (SS) heart rate will be recorded. The formula for estimated VO2max is:

Estimated VO2max (ml·kg^-1^·min^-1^) = 15.1 + 21.8 (speed in mph) – 0.327 (SS heart rate in bpm) – 0.263 (speed x age) + 0.00504 (SS heart rate x age) + 5.98 (gender; female = 0, male = 1).

## **9.9 Questionnaires**

The following questionnaires will be administered:

The Physical Activity Readiness Questionnaire (PAR-Q) will be administered for eligibility screening to determine whether potential participants are healthy enough and have sufficient baseline fitness levels for exercise training. If a potential participant answers “yes” to any questions on the PAR-Q, they will be encouraged to check with their doctor before becoming much more physically active, and will be required to receive medical clearance from a physician to participate in this study.

The Mini-Mental State Examination (MMSE) is the most commonly applied test to screen for cognitive deficits [114]. The MMSE will be administered for eligibility screening to determine the mental status of potential participants. The maximum score is 30. A score of 23 or lower is indicative of cognitive impairment, and will preclude the patient from participating in this study. These patients will be referred to a clinician with experience in dementia assessment.

Physical activity levels will be assessed using the International Physical Activity Questionnaire (IPAQ), administered at baseline, pre-intervention, and post-intervention. The IPAQ has been validated in adults over the age of 18 with varying activity levels across 12 countries [115]. The IPAQ aims to identify physical activity and exercise habits, and will be used to determine the average volume of activity over 1 week in metabolic equivalents (METs).

Quality of life (QOL) is an important domain for dementia assessment [1]. Increased physical activity can improve QOL in healthy older adults [116, 117], frail older adults [118], and patients with mild cognitive impairment [119]. To test the effects of the RT intervention on QOL, the Satisfaction With Life Scale (SWLS) will be administered at baseline, pre-intervention, and post-intervention [120]. Research using the SWLS suggests that it is sensitive enough to detect changes in life satisfaction over time [121]. In addition, general health-related quality of life status will be assessed using the Short Form-36 (SF-36) [122].

Sleep is an important consideration for cognitive performance in older adults [123]. Poor sleep is associated with impaired cognitive function [124, 125]. The Pittsburgh Sleep Quality Index (PSQI) will be administered at baseline, pre-intervention, and post-intervention to evaluate sleep quality. The clinemetric properties of the PSQI have made it a standard assessment for clinical research in older adults [126-128].

Social interaction and engagement are important confounding variables in intervention studies targeting cognitive health [34, 129]. The Interpersonal Support Evaluation List (ISEL) is a validated measure of perceptions of social support [130] that will help us understand whether the effectiveness of our intervention is modified by perceptions of social support, i.e. to control for the influence of social interaction during personal one-on-one exercise training.

## **9.10 Study Calendar**

|  | Week # | Day 1 | Day 2 | Day 3 |
| --- | --- | --- | --- | --- |
|  | 0 | Informed Consent  Eligibility |  |  |
| Baseline Tests | 1-2 | **Testing Part 1:**  Cognitive Testing  QOL Questionnaires  PSQI  MRI Scan | **Familiarization Day:**  Descriptive Factors  Exercise Familiarization | **Testing Part 2:**  IPAQ  Blood Pressure  Body Composition  Blood Draw  Physical Function  Muscle Strength  Estimated VO2max |
| Control | 3-14 | 12-week Control Period | | |
| Pre Tests | 15 | **Testing Part 1** | **Testing Part 2** |  |
| RT | 16-27 | 12-week RT Intervention | | |
| Buffer Period  (if necessary) | 28-29 | Missed Session Completion | | |
| Post Tests | 28-29, 29-30, or  30-31 | **Testing Part 1** | **Testing Part 2** |  |

# **10.0 Criteria for Evaluation and Endpoint Definitions**

The outcome status of all eligible patients will be reported. Endpoint definitions include completion of the resistance training program with at least 80% of the sessions attended and baseline and post-intervention measurements for all variables.

# **11.0 Special Instructions**

N/A

# **12.0 Data Collection and Monitoring**

All data will be de-identified to prevent participants’ identities from being connected with their information. Each participant will receive a Patient Identification number. Data will be stored in a locked file cabinet in an office (CHP-149). All data will be inputted into a digital spreadsheet for subsequent use/analyses. Only members of the study team will have access to the digital spreadsheets.

# **13.0 Statistical Considerations**

## **13.1 Statistical Analysis Plan**

Participant characteristics will be described at baseline. Continuous variables will be examined for normality and described by either means and standard deviations or medians and interquartile range (if not normal); categorical variables will be described with N (%) of each category. Participant characteristics will be compared with missing data and attrition to determine if there are any systematic patterns that should be adjusted for in secondary analyses; any difference with p < 0.25 will be used in secondary analyses to see if there is an effect on the intent-to-treat model.

Comparison of cerebrovascular function (Hypothesis 1) and cognition (Hypothesis 2) between groups will follow intent-to-treat models using longitudinal linear mixed effects modeling to assess the change in these outcomes, with the main factor of period – control versus RT. The stratifying factor of gender will also be included in the model, as is standard. This same modeling will be used to test whether cerebrovascular function mediates the effect of group assignment on cognition by adding cerebrovascular function to the model of cognition and examining the change in the beta coefficient of group (exploratory objective 1). A change in beta coefficient of >20%, will be considered indicative of mediation. Because these data will be preliminary, the primary endpoint will be the effect size calculated from the estimated marginal means of differences in the change by period from the models. This can then be translated more directly into potential clinical effect.

This same modeling technique will be used for outcomes defined in exploratory objective 2. To assess the association of changes in cerebrovascular function and cognition with other outcomes (exploratory objective 3), Spearman correlations of change in outcomes, adjusted for baseline level will be computed for the total group, and stratified by group assignment.

All analyses will be performed with SPSS (v.24).

## **13.2 Sample Size and Power Considerations**

Because this is a proof-of-concept clinical trial, sample size will be determined based on feasibility and study funding. Previous investigations on CBF after 12 weeks of aerobic exercise [47] suggest that 20 participants per group is sufficient to determine response variance and effect sizes to establish preliminary signals of efficacy. Attrition rate is anticipated to be 15%, a conservative estimate, as the attrition rate of previous RT interventions of similar length (12 weeks, 3days/week) in healthy older adults is about 5.7% [131]. Our previous RT intervention in older adult men, upon which the current study’s RT program is based, had no dropouts due to injury, illness, or personal choice [80]. Accounting for 15% attrition (~3) yields a final, proposed sample size of 23.

Sensitivity analysis using these parameters was performed in G*Power 3.1.9.2. With the proposed sample size of 23, we would have sufficient power to statistically detect a large effect size d between dependent means of 0.611 with α = 0.05 and 1-β = 0.80.

# **14.0 Registration Guidelines**

Prospective participants may contact investigators of the study at the CERC located in the Center for Health Professions (CHP149) on the Health Sciences Campus of the University of Southern California, or may call the lab at 323-442-2180. Registration details and additional information will be provided to all prospective participants. All prospective study participants will be required to fill out an informed consent prior to beginning study requirements. Investigators will register patients to the study after the informed consent has been obtained, then participants will be screened to ensure eligibility in accordance with investigation requirements.

Note: At the time of registration, two copies of a signed and dated patient Informed Consent form with Bill of Rights will be available (one copy for the patient; and the other for the PI’s file).

# **15.0 Biohazard Containment**

All blood draws will be performed by a California-State Licensed phlebotomist in a private room designated for blood draws at the CERC. Waste from blood sampling procedures will be handled using universal precautions (e.g., needles/syringes placed in sharps containers, dry waste contaminated with human blood placed in biomedical waste containers).

# **16.0 Ethical and Regulatory Considerations**

All institutional and Federal regulations concerning the Informed Consent form will be fulfilled. The study will be conducted in adherence to ICH Good Clinical Practice.

## **16.1 Risk/Benefit Assessment:**

Potential risks: Participants might experience exercise-related fatigue, dizziness, nausea, strains, and/or soreness. All participants will be supervised through proper warm-up, cool-down, and exercise instruction by an exercise specialist to minimize these discomforts.

There is a chance the subject may experience slight discomfort or nausea during the blood draw. Immediate side effects may include pain or discomfort associated with needle stick as well as possibility of bruising and nerve aggravation at the blood draw site in the antecubital space of the arm. The risks of two blood draws (baseline and post-intervention) are greater than minimal. However, the amount of blood (~20mL per draw) that will be taken per blood draw at only one site on the participant’s body is small.

Potential benefits: There are no immediate benefits to the subject. However, the participants will have the benefit of learning about resistance training program design, their physical fitness, and physical fitness testing, as well as experiencing improvements in overall health due to an exercise intervention. Potential study participants are encouraged to participate if they are interested in research and to further their understanding of resistance training, physical fitness and brain health.

## **16.2 Financial Obligations and Compensation**

There are no financial obligations on the part of the subject. There will be no financial compensation for participation in this study. However, participants will receive supervised RT or flexibility training by a certified exercise specialist.

# **17.0 References**

[1] H. Bárrios, S. Narciso, M. Guerreiro, J. Maroco, R. Logsdon, A. De Mendonça, Quality of life in patients with mild cognitive impairment, Aging & mental health 17(3) (2013) 287-292.

[2] A. Alzheimer’s, 2015 Alzheimer's disease facts and figures, Alzheimer's & dementia: the journal of the Alzheimer's Association 11(3) (2015) 332.

[3] M.D. Hurd, P. Martorell, A. Delavande, K.J. Mullen, K.M. Langa, Monetary costs of dementia in the United States, New England Journal of Medicine 368(14) (2013) 1326-1334.

[4] M. Prince, R. Bryce, E. Albanese, A. Wimo, W. Ribeiro, C.P. Ferri, The global prevalence of dementia: a systematic review and metaanalysis, Alzheimer's & Dementia 9(1) (2013) 63-75. e2.

[5] G.M. McKhann, D.S. Knopman, H. Chertkow, B.T. Hyman, C.R. Jack, C.H. Kawas, W.E. Klunk, W.J. Koroshetz, J.J. Manly, R. Mayeux, The diagnosis of dementia due to Alzheimer’s disease: Recommendations from the National Institute on Aging-Alzheimer’s Association workgroups on diagnostic guidelines for Alzheimer's disease, Alzheimer's & dementia 7(3) (2011) 263-269.

[6] H.C. Kales, L.N. Gitlin, C.G. Lyketsos, Assessment and management of behavioral and psychological symptoms of dementia, bmj 350(7) (2015) h369.

[7] E.B. Fauth, S. Schwartz, J.T. Tschanz, T. Østbye, C. Corcoran, M.C. Norton, Baseline disability in activities of daily living predicts dementia risk even after controlling for baseline global cognitive ability and depressive symptoms, International journal of geriatric psychiatry 28(6) (2013) 597-606.

[8] H. Liu-Seifert, E. Siemers, K. Sundell, K. Price, B. Han, K. Selzler, P. Aisen, J. Cummings, J. Raskin, R. Mohs, Cognitive and functional decline and their relationship in patients with mild Alzheimer's dementia, Journal of Alzheimer's Disease 43(3) (2015) 949-955.

[9] R. Schulz, T.L. Patterson, Caregiving in geriatric psychiatry, The American Journal of Geriatric Psychiatry 12(3) (2004) 234-237.

[10] B.A. Springate, G. Tremont, Dimensions of caregiver burden in dementia: impact of demographic, mood, and care recipient variables, The American Journal of Geriatric Psychiatry 22(3) (2014) 294-300.

[11] C.E. Munro, N.J. Donovan, B.J. Guercio, S.E. Wigman, A.P. Schultz, R.E. Amariglio, D.M. Rentz, K.A. Johnson, R.A. Sperling, G.A. Marshall, Neuropsychiatric symptoms and functional connectivity in mild cognitive impairment, Journal of Alzheimer's Disease 46(3) (2015) 727-735.

[12] M.S. Albert, S.T. DeKosky, D. Dickson, B. Dubois, H.H. Feldman, N.C. Fox, A. Gamst, D.M. Holtzman, W.J. Jagust, R.C. Petersen, The diagnosis of mild cognitive impairment due to Alzheimer’s disease: Recommendations from the National Institute on Aging-Alzheimer’s Association workgroups on diagnostic guidelines for Alzheimer's disease, Alzheimer's & dementia 7(3) (2011) 270-279.

[13] B. Winblad, K. Palmer, M. Kivipelto, V. Jelic, L. Fratiglioni, L.O. Wahlund, A. Nordberg, L. Bäckman, M. Albert, O. Almkvist, Mild cognitive impairment–beyond controversies, towards a consensus: report of the International Working Group on Mild Cognitive Impairment, Journal of internal medicine 256(3) (2004) 240-246.

[14] N.J. Donovan, R.E. Amariglio, A.S. Zoller, R.K. Rudel, T. Gomez-Isla, D. Blacker, B.T. Hyman, J.J. Locascio, K.A. Johnson, R.A. Sperling, Subjective cognitive concerns and neuropsychiatric predictors of progression to the early clinical stages of Alzheimer disease, The American Journal of Geriatric Psychiatry 22(12) (2014) 1642-1651.

[15] K. Deckers, M.P. Boxtel, O.J. Schiepers, M. Vugt, J.L. Muñoz Sánchez, K.J. Anstey, C. Brayne, J.F. Dartigues, K. Engedal, M. Kivipelto, Target risk factors for dementia prevention: a systematic review and Delphi consensus study on the evidence from observational studies, International journal of geriatric psychiatry 30(3) (2015) 234-246.

[16] E.L. Abner, R.J. Kryscio, G.E. Cooper, D.W. Fardo, G.A. Jicha, M.S. Mendiondo, P.T. Nelson, C.D. Smith, L.J. Van Eldik, L. Wan, Mild cognitive impairment: statistical models of transition using longitudinal clinical data, International journal of Alzheimer’s disease 2012 (2012).

[17] J. Tschanz, K. Welsh-Bohmer, C. Lyketsos, C. Corcoran, R.C. Green, K. Hayden, M. Norton, P. Zandi, L. Toone, N. West, Conversion to dementia from mild cognitive disorder The Cache County Study, Neurology 67(2) (2006) 229-234.

[18] J. Ngo, J.M. Holroyd-Leduc, Systematic review of recent dementia practice guidelines, Age Ageing 44(1) (2014) 25-33.

[19] G. Odenheimer, S. Borson, A.E. Sanders, R.J. Swain-Eng, H.H. Kyomen, S. Tierney, L.N. Gitlin, M.A. Forciea, J. Absher, J. Shega, Quality improvement in neurology Dementia management quality measures, Neurology 81(17) (2013) 1545-1549.

[20] E. Loveman, C. Green, J. Kirby, A. Takeda, J. Picot, E. Payne, A. Clegg, The clinical and cost-effectiveness of donepezil, rivastigmine, galantamine and memantine for Alzheimer's disease, (2006).

[21] C.R. Jack, R.C. Petersen, M. Grundman, S. Jin, A. Gamst, C.P. Ward, D. Sencakova, R.S. Doody, L.J. Thal, A.s.D.C. Study, Longitudinal MRI findings from the vitamin E and donepezil treatment study for MCI, Neurobiology of aging 29(9) (2008) 1285-1295.

[22] C.W. Ritchie, G.M. Terrera, T.J. Quinn, Dementia trials and dementia tribulations: methodological and analytical challenges in dementia research, Alzheimer's research & therapy 7(1) (2015) 31.

[23] C.R. Jack, D.S. Knopman, W.J. Jagust, L.M. Shaw, P.S. Aisen, M.W. Weiner, R.C. Petersen, J.Q. Trojanowski, Hypothetical model of dynamic biomarkers of the Alzheimer's pathological cascade, The Lancet Neurology 9(1) (2010) 119-128.

[24] P.J. Snyder, C.E. Jackson, R.C. Petersen, A.S. Khachaturian, J. Kaye, M.S. Albert, S. Weintraub, Assessment of cognition in mild cognitive impairment: a comparative study, Alzheimer's & Dementia 7(3) (2011) 338-355.

[25] J.L. Cummings, R. Doody, C. Clark, Disease-modifying therapies for Alzheimer disease Challenges to early intervention, Neurology 69(16) (2007) 1622-1634.

[26] M. Dichgans, D. Leys, Vascular cognitive impairment, Circulation research 120(3) (2017) 573-591.

[27] P. Gorelick, A. Scuteri, S. Black, C. Decarli, S. Greenberg, C. Iadecola, L. Launer, S. Laurent, O. Lopez, D. Nyenhuis, American Heart Association Stroke Council, Council on Epidemiology and Prevention, Council on Cardiovascular Nursing, Council on Cardiovascular Radiology and Intervention, and Council on Cardiovascular Surgery and Anesthesia. Vascular contributions to cognitive impairment and dementia: a statement for healthcare professionals from the american heart association/american stroke association, Stroke 42(9) (2011) 2672-2713.

[28] K. Rockwood, C. Wentzel, V. Hachinski, D. Hogan, C. MacKnight, I. McDowell, Prevalence and outcomes of vascular cognitive impairment, Neurology 54(2) (2000) 447-447.

[29] R.A. Corriveau, F. Bosetti, M. Emr, J.T. Gladman, J.I. Koenig, C.S. Moy, K. Pahigiannis, S.P. Waddy, W. Koroshetz, The science of vascular contributions to cognitive impairment and dementia (VCID): a framework for advancing research priorities in the cerebrovascular biology of cognitive decline, Cellular and molecular neurobiology 36(2) (2016) 281-288.

[30] A. Montagne, D.A. Nation, J. Pa, M.D. Sweeney, A.W. Toga, B.V. Zlokovic, Brain imaging of neurovascular dysfunction in Alzheimer’s disease, Acta neuropathologica 131(5) (2016) 687-707.

[31] A. Popa-Wagner, A.-M. Buga, B. Popescu, D. Muresanu, Vascular cognitive impairment, dementia, aging and energy demand. A vicious cycle, Journal of neural transmission 122(1) (2015) 47-54.

[32] G.C. Román, Vascular dementia: distinguishing characteristics, treatment, and prevention, J Am Geriatr Soc 51(5s2) (2003).

[33] M. Dichgans, V. Zietemann, Prevention of vascular cognitive impairment, Stroke 43(11) (2012) 3137-3146.

[34] T. Liu-Ambrose, J.R. Best, Exercise is Medicine for the Aging Brain, Kinesiology Review 6(1) (2017) 22-29.

[35] M. Hamer, Y. Chida, Physical activity and risk of neurodegenerative disease: a systematic review of prospective evidence, Psychological medicine 39(1) (2009) 3-11.

[36] F. Sofi, D. Valecchi, D. Bacci, R. Abbate, G.F. Gensini, A. Casini, C. Macchi, Physical activity and risk of cognitive decline: a meta‐analysis of prospective studies, Journal of internal medicine 269(1) (2011) 107-117.

[37] D.E. Barnes, K. Yaffe, The projected effect of risk factor reduction on Alzheimer's disease prevalence, The Lancet Neurology 10(9) (2011) 819-828.

[38] C.K. Barha, L.A. Galea, L.S. Nagamatsu, K.I. Erickson, T. Liu-Ambrose, Personalising exercise recommendations for brain health: considerations and future directions, Br J Sports Med 51(8) (2017) 636-639.

[39] W.J. Chodzko-Zajko, D.N. Proctor, M.A.F. Singh, C.T. Minson, C.R. Nigg, G.J. Salem, J.S. Skinner, Exercise and physical activity for older adults, Medicine & science in sports & exercise 41(7) (2009) 1510-1530.

[40] S.J. Colcombe, K.I. Erickson, P.E. Scalf, J.S. Kim, R. Prakash, E. McAuley, S. Elavsky, D.X. Marquez, L. Hu, A.F. Kramer, Aerobic exercise training increases brain volume in aging humans, The Journals of Gerontology Series A: Biological Sciences and Medical Sciences 61(11) (2006) 1166-1170.

[41] S. Colcombe, A.F. Kramer, Fitness effects on the cognitive function of older adults: a meta-analytic study, Psychological science 14(2) (2003) 125-130.

[42] K.I. Erickson, M.W. Voss, R.S. Prakash, C. Basak, A. Szabo, L. Chaddock, J.S. Kim, S. Heo, H. Alves, S.M. White, Exercise training increases size of hippocampus and improves memory, Proceedings of the National Academy of Sciences 108(7) (2011) 3017-3022.

[43] B.V. Zlokovic, Neurovascular pathways to neurodegeneration in Alzheimer's disease and other disorders, Nature Reviews Neuroscience 12(12) (2011) 723-738.

[44] J.N. Barnes, Exercise, cognitive function, and aging, Advances in physiology education 39(2) (2015) 55-62.

[45] E. Bullitt, F. Rahman, J. Smith, E. Kim, D. Zeng, L. Katz, B. Marks, The effect of exercise on the cerebral vasculature of healthy aged subjects as visualized by MR angiography, American Journal of Neuroradiology 30(10) (2009) 1857-1863.

[46] A.C. Pereira, D.E. Huddleston, A.M. Brickman, A.A. Sosunov, R. Hen, G.M. McKhann, R. Sloan, F.H. Gage, T.R. Brown, S.A. Small, An in vivo correlate of exercise-induced neurogenesis in the adult dentate gyrus, Proceedings of the National Academy of Sciences 104(13) (2007) 5638-5643.

[47] A. Maass, S. Düzel, M. Goerke, A. Becke, U. Sobieray, K. Neumann, M. Lövdén, U. Lindenberger, L. Bäckman, R. Braun-Dullaeus, Vascular hippocampal plasticity after aerobic exercise in older adults, Molecular psychiatry 20(5) (2015) 585-593.

[48] B. Sabayan, R.G. Westendorp, J. van der Grond, D.J. Stott, N. Sattar, M.J. van Osch, M.A. van Buchem, A.J. de Craen, Markers of endothelial dysfunction and cerebral blood flow in older adults, Neurobiology of aging 35(2) (2014) 373-377.

[49] G.V. Mendonca, P. Pezarat-Correia, J.R. Vaz, L. Silva, K.S. Heffernan, Impact of aging on endurance and neuromuscular physical performance: The role of vascular senescence, Sports Medicine 47(4) (2017) 583-598.

[50] M.D. Peterson, A. Sen, P.M. Gordon, Influence of resistance exercise on lean body mass in aging adults: a meta-analysis, Medicine and science in sports and exercise 43(2) (2011) 249.

[51] H. Nakamoto, Y. Yoshitake, Y. Takai, H. Kanehisa, T. Kitamura, M. Kawanishi, S. Mori, Knee extensor strength is associated with Mini-Mental State Examination scores in elderly men, European journal of applied physiology 112(5) (2012) 1945-1953.

[52] R. Pedrero‐Chamizo, U. Albers, J.L. Tobaruela, A. Meléndez, M.J. Castillo, M. González‐Gross, Physical strength is associated with Mini‐Mental State Examination scores in Spanish institutionalized elderly, Geriatrics & gerontology international 13(4) (2013) 1026-1034.

[53] E.G. Artero, D.-c. Lee, C.J. Lavie, V. España-Romero, X. Sui, T.S. Church, S.N. Blair, Effects of muscular strength on cardiovascular risk factors and prognosis, Journal of cardiopulmonary rehabilitation and prevention 32(6) (2012) 351.

[54] M. Ochi, K. Kohara, Y. Tabara, T. Kido, E. Uetani, N. Ochi, M. Igase, T. Miki, Arterial stiffness is associated with low thigh muscle mass in middle-aged to elderly men, Atherosclerosis 212(1) (2010) 327-332.

[55] X. Xu, B.A. Jerskey, D.M. Cote, E.G. Walsh, J.J. Hassenstab, M.E. Ladino, U.S. Clark, D.R. Labbe, J.J. Gunstad, A. Poppas, Cerebrovascular perfusion among older adults is moderated by strength training and gender, Neuroscience letters 560 (2014) 26-30.

[56] R.C. Cassilhas, V.A. Viana, V. Grassmann, R.T. Santos, R.F. Santos, S. Tufik, M.T. Mello, The impact of resistance exercise on the cognitive function of the elderly, Medicine and science in sports and exercise 39(8) (2007) 1401.

[57] T. Liu-Ambrose, L.S. Nagamatsu, M.W. Voss, K.M. Khan, T.C. Handy, Resistance training and functional plasticity of the aging brain: a 12-month randomized controlled trial, Neurobiology of aging 33(8) (2012) 1690-1698.

[58] M.A.F. Singh, N. Gates, N. Saigal, G.C. Wilson, J. Meiklejohn, H. Brodaty, W. Wen, N. Singh, B.T. Baune, C. Suo, The Study of Mental and Resistance Training (SMART) study—resistance training and/or cognitive training in mild cognitive impairment: a randomized, double-blind, double-sham controlled trial, Journal of the American Medical Directors Association 15(12) (2014) 873-880.

[59] M.A. Williams, W.L. Haskell, P.A. Ades, E.A. Amsterdam, V. Bittner, B.A. Franklin, M. Gulanick, S.T. Laing, K.J. Stewart, Resistance exercise in individuals with and without cardiovascular disease: 2007 update, Circulation 116(5) (2007) 572-584.

[60] A.W. Ashor, J. Lara, M. Siervo, C. Celis-Morales, C. Oggioni, D.G. Jakovljevic, J.C. Mathers, Exercise modalities and endothelial function: a systematic review and dose–response meta-analysis of randomized controlled trials, Sports medicine 45(2) (2015) 279-296.

[61] A.L. Spence, H.H. Carter, L.H. Naylor, D.J. Green, A prospective randomized longitudinal study involving 6 months of endurance or resistance exercise. Conduit artery adaptation in humans, The Journal of physiology 591(5) (2013) 1265-1275.

[62] Y. Mavros, N. Gates, G.C. Wilson, N. Jain, J. Meiklejohn, H. Brodaty, W. Wen, N. Singh, B.T. Baune, C. Suo, Mediation of cognitive function improvements by strength gains after resistance training in older adults with mild cognitive impairment: outcomes of the study of mental and resistance training, J Am Geriatr Soc 65(3) (2017) 550-559.

[63] R. Forte, C.A. Boreham, J.C. Leite, G. De Vito, L. Brennan, E.R. Gibney, C. Pesce, Enhancing cognitive functioning in the elderly: multicomponent vs resistance training, Clinical interventions in aging 8 (2013) 19.

[64] P.A. Boyle, A.S. Buchman, R.S. Wilson, S.E. Leurgans, D.A. Bennett, Association of muscle strength with the risk of Alzheimer disease and the rate of cognitive decline in community-dwelling older persons, Archives of neurology 66(11) (2009) 1339-1344.

[65] A. Kandola, J. Hendrikse, P.J. Lucassen, M. Yücel, Aerobic exercise as a tool to improve hippocampal plasticity and function in humans: practical implications for mental health treatment, Frontiers in human neuroscience 10 (2016).

[66] C. Suo, M.F. Singh, N. Gates, W. Wen, P. Sachdev, H. Brodaty, N. Saigal, G.C. Wilson, J. Meiklejohn, N. Singh, Therapeutically relevant structural and functional mechanisms triggered by physical and cognitive exercise, Molecular psychiatry (2016).

[67] N. Bolandzadeh, R. Tam, T.C. Handy, L.S. Nagamatsu, C.L. Hsu, J.C. Davis, E. Dao, B.L. Beattie, T. Liu‐Ambrose, Resistance Training and White Matter Lesion Progression in Older Women: Exploratory Analysis of a 12‐Month Randomized Controlled Trial, J Am Geriatr Soc 63(10) (2015) 2052-2060.

[68] R. Cassilhas, K. Lee, J. Fernandes, M. Oliveira, S. Tufik, R. Meeusen, M. De Mello, Spatial memory is improved by aerobic and resistance exercise through divergent molecular mechanisms, Neuroscience 202 (2012) 309-317.

[69] M. Murer, Q. Yan, R. Raisman-Vozari, Brain-derived neurotrophic factor in the control human brain, and in Alzheimer’s disease and Parkinson’s disease, Progress in neurobiology 63(1) (2001) 71-124.

[70] F. Coelho, D. Pereira, L. Lustosa, J. Silva, J. Dias, R. Dias, B. Queiroz, A. Teixeira, M. Teixeira, L. Pereira, Physical therapy intervention (PTI) increases plasma brain-derived neurotrophic factor (BDNF) levels in non-frail and pre-frail elderly women, Archives of gerontology and geriatrics 54(3) (2012) 415-420.

[71] F.G. de Melo Coelho, S. Gobbi, C.A.A. Andreatto, D.I. Corazza, R.V. Pedroso, R.F. Santos-Galduróz, Physical exercise modulates peripheral levels of brain-derived neurotrophic factor (BDNF): a systematic review of experimental studies in the elderly, Archives of gerontology and geriatrics 56(1) (2013) 10-15.

[72] K.A. Intlekofer, N.C. Berchtold, M. Malvaez, A.J. Carlos, S.C. McQuown, M.J. Cunningham, M.A. Wood, C.W. Cotman, Exercise and sodium butyrate transform a subthreshold learning event into long-term memory via a brain-derived neurotrophic factor-dependent mechanism, Neuropsychopharmacology 38(10) (2013) 2027.

[73] C.W. Cotman, N.C. Berchtold, L.-A. Christie, Exercise builds brain health: key roles of growth factor cascades and inflammation, Trends in neurosciences 30(9) (2007) 464-472.

[74] K. Fabel, K. Fabel, B. Tam, D. Kaufer, A. Baiker, N. Simmons, C.J. Kuo, T.D. Palmer, VEGF is necessary for exercise‐induced adult hippocampal neurogenesis, European Journal of Neuroscience 18(10) (2003) 2803-2812.

[75] M.W. Voss, K.I. Erickson, R.S. Prakash, L. Chaddock, J.S. Kim, H. Alves, A. Szabo, S.M. Phillips, T.R. Wójcicki, E.L. Mailey, Neurobiological markers of exercise-related brain plasticity in older adults, Brain, behavior, and immunity 28 (2013) 90-99.

[76] A. Maass, S. Düzel, T. Brigadski, M. Goerke, A. Becke, U. Sobieray, K. Neumann, M. Lövdén, U. Lindenberger, L. Bäckman, Relationships of peripheral IGF-1, VEGF and BDNF levels to exercise-related changes in memory, hippocampal perfusion and volumes in older adults, Neuroimage 131 (2016) 142-154.

[77] R.G. de Souza Vale, R.D. de Oliveira, C.S. Pernambuco, J. da Silva Novaes, A.d.F.D. de Andrade, Effects of muscle strength and aerobic training on basal serum levels of IGF-1 and cortisol in elderly women, Archives of gerontology and geriatrics 49(3) (2009) 343-347.

[78] S.J. Fleck, W. Kraemer, Designing Resistance Training Programs, 4E, Human Kinetics2014.

[79] G.G. Haff, N.T. Triplett, Essentials of Strength Training and Conditioning 4th Edition, Human kinetics2015.

[80] M.G. Villanueva, C.J. Lane, E.T. Schroeder, Short rest interval lengths between sets optimally enhance body composition and performance with 8 weeks of strength resistance training in older men, European journal of applied physiology 115(2) (2015) 295-308.

[81] M.G. Villanueva, M.G. Villanueva, C.J. Lane, E.T. Schroeder, Influence of rest interval length on acute testosterone and cortisol responses to volume-load equated total body hypertrophic and strength protocols, Journal of strength and conditioning research/National Strength & Conditioning Association 26(10) (2012) 2755.

[82] J.L. Kiwata, T.B. Dorff, E. Schroeder, C.M. Dieli-Conwright, Effect of a supervised exercise intervention on sarcopenic obesity and metabolic syndrome in prostate cancer patients: A randomized pilot study, AACR, 2017.

[83] J. Pa, S. Skinner, P. Wais, C. Thompson, A. Gazzaley, Exercise to enhance hippocampal function and memory in older adults: Proof of concept findings from a 12-week exercise intervention trial, Alzheimer's & Dementia: The Journal of the Alzheimer's Association 11(7) (2015) P95-P96.

[84] L. Yan, C.Y. Liu, R.X. Smith, M. Jog, M. Langham, K. Krasileva, Y. Chen, J.M. Ringman, D.J. Wang, Assessing intracranial vascular compliance using dynamic arterial spin labeling, NeuroImage 124 (2016) 433-441.

[85] L. Yan, C. Li, E. Kilroy, F.W. Wehrli, D.J. Wang, Quantification of arterial cerebral blood volume using multiphase‐balanced SSFP‐based ASL, Magnetic resonance in medicine 68(1) (2012) 130-139.

[86] D.J. Wang, J.R. Alger, J.X. Qiao, M. Gunther, W.B. Pope, J.L. Saver, N. Salamon, D.S. Liebeskind, U.S. Investigators, Multi-delay multi-parametric arterial spin-labeled perfusion MRI in acute ischemic stroke—comparison with dynamic susceptibility contrast enhanced perfusion imaging, NeuroImage: Clinical 3 (2013) 1-7.

[87] B. MacIntosh, W. Swardfager, A. Robertson, E. Tchistiakova, M. Saleem, P. Oh, N. Herrmann, B. Stefanovic, K. Lanctôt, Regional cerebral arterial transit time hemodynamics correlate with vascular risk factors and cognitive function in men with coronary artery disease, American Journal of Neuroradiology 36(2) (2015) 295-301.

[88] J.R. Best, B.K. Chiu, C.L. Hsu, L.S. Nagamatsu, T. Liu-Ambrose, Long-term effects of resistance exercise training on cognition and brain volume in older women: results from a randomized controlled trial, Journal of the international neuropsychological society 21(10) (2015) 745-756.

[89] P. Peig-Chiello, W.J. Perrig, R. Ehrsam, H.B. Staehelin, F. Krings, The effects of resistance training on well-being and memory in elderly volunteers, Age Ageing 27(4) (1998) 469-475.

[90] M.D. Greicius, G. Srivastava, A.L. Reiss, V. Menon, Default-mode network activity distinguishes Alzheimer's disease from healthy aging: evidence from functional MRI, Proceedings of the National Academy of Sciences of the United States of America 101(13) (2004) 4637-4642.

[91] M. Havlík, From Anomalies to Essential Scientific Revolution? Intrinsic Brain Activity in the Light of Kuhn's Philosophy of Science, Frontiers in Systems Neuroscience 11 (2017).

[92] X. Golay, J. Hendrikse, T.C. Lim, Perfusion imaging using arterial spin labeling, Topics in Magnetic Resonance Imaging 15(1) (2004) 10-27.

[93] J.A. Detre, J. Wang, Z. Wang, H. Rao, Arterial spin-labeled perfusion MRI in basic and clinical neuroscience, Current opinion in neurology 22(4) (2009) 348-355.

[94] S. Weintraub, S.S. Dikmen, R.K. Heaton, D.S. Tulsky, P.D. Zelazo, P.J. Bauer, N.E. Carlozzi, J. Slotkin, D. Blitz, K. Wallner-Allen, Cognition assessment using the NIH Toolbox, Neurology 80(11 Supplement 3) (2013) S54-S64.

[95] S. Weintraub, S.S. Dikmen, R.K. Heaton, D.S. Tulsky, P.D. Zelazo, J. Slotkin, N.E. Carlozzi, P.J. Bauer, K. Wallner-Allen, N. Fox, The cognition battery of the NIH toolbox for assessment of neurological and behavioral function: Validation in an adult sample, Journal of the International Neuropsychological Society 20(6) (2014) 567-578.

[96] R.K. Heaton, N. Akshoomoff, D. Tulsky, D. Mungas, S. Weintraub, S. Dikmen, J. Beaumont, K.B. Casaletto, K. Conway, J. Slotkin, Reliability and validity of composite scores from the NIH Toolbox Cognition Battery in adults, Journal of the International Neuropsychological Society 20(6) (2014) 588-598.

[97] T.E. Goldberg, P.D. Harvey, K.A. Wesnes, P.J. Snyder, L.S. Schneider, Practice effects due to serial cognitive assessment: implications for preclinical Alzheimer's disease randomized controlled trials, Alzheimer's & Dementia: Diagnosis, Assessment & Disease Monitoring 1(1) (2015) 103-111.

[98] M.A. do Nascimento, R.S.B. Januário, A.M. Gerage, J.L. Mayhew, F.L.C. Pina, E.S. Cyrino, Familiarization and reliability of one repetition maximum strength testing in older women, The Journal of Strength & Conditioning Research 27(6) (2013) 1636-1642.

[99] M. Brzycki, Strength testing—predicting a one-rep max from reps-to-fatigue, Journal of Physical Education, Recreation & Dance 64(1) (1993) 88-90.

[100] K.M. Knutzen, L.R. Brilla, D. Caine, Validity of 1RM Prediction Equations for Older Adults, The Journal of Strength & Conditioning Research 13(3) (1999) 242-246.

[101] L.B. Verdijk, L. Van Loon, K. Meijer, H.H. Savelberg, One-repetition maximum strength test represents a valid means to assess leg strength in vivo in humans, Journal of sports sciences 27(1) (2009) 59-68.

[102] S. Studenski, S. Perera, K. Patel, C. Rosano, K. Faulkner, M. Inzitari, J. Brach, J. Chandler, P. Cawthon, E.B. Connor, Gait speed and survival in older adults, Jama 305(1) (2011) 50-58.

[103] R. Margaria, P. Aghemo, E. Rovelli, Measurement of muscular power (anaerobic) in man, Journal of applied physiology 21(5) (1966) 1662-1664.

[104] D. Podsiadlo, S. Richardson, The timed “Up & Go”: a test of basic functional mobility for frail elderly persons, J Am Geriatr Soc 39(2) (1991) 142-148.

[105] G.F. Coughlan, K. Fullam, E. Delahunt, C. Gissane, B.M. Caulfield, A comparison between performance on selected directions of the star excursion balance test and the Y balance test, Journal of athletic training 47(4) (2012) 366-371.

[106] T.M. Steffen, T.A. Hacker, L. Mollinger, Age-and gender-related test performance in community-dwelling elderly people: Six-Minute Walk Test, Berg Balance Scale, Timed Up & Go Test, and gait speeds, Physical therapy 82(2) (2002) 128-137.

[107] M. vaniersel, C.M. Benraad, M.M. Olderikkert, Validity and reliability of quantitative gait analysis in geriatric patients with and without dementia, J Am Geriatr Soc 55(4) (2007) 632-633.

[108] J.D. Ries, J.L. Echternach, L. Nof, M. Gagnon Blodgett, Test-retest reliability and minimal detectable change scores for the timed “up & go” test, the six-minute walk test, and gait speed in people with Alzheimer disease, Physical therapy 89(6) (2009) 569-579.

[109] J.F. Bean, D.K. Kiely, S. LaRose, J. Alian, W.R. Frontera, Is stair climb power a clinically relevant measure of leg power impairments in at-risk older adults?, Archives of physical medicine and rehabilitation 88(5) (2007) 604-609.

[110] T. Bhatt, D. Espy, F. Yang, Y.-C. Pai, Dynamic gait stability, clinical correlates, and prognosis of falls among community-dwelling older adults, Archives of physical medicine and rehabilitation 92(5) (2011) 799-805.

[111] A. Shumway-Cook, S. Brauer, M. Woollacott, Predicting the probability for falls in community-dwelling older adults using the Timed Up & Go Test, Physical therapy 80(9) (2000) 896-903.

[112] P.A. Gribble, J. Hertel, P. Plisky, Using the Star Excursion Balance Test to assess dynamic postural-control deficits and outcomes in lower extremity injury: a literature and systematic review, Journal of athletic training 47(3) (2012) 339-357.

[113] C.B. Ebbeling, A. Ward, E.M. Puleo, J. Widrick, J.M. Rippe, Development of a single-stage submaximal treadmill walking test, Medicine and science in sports and exercise 23(8) (1991) 966-973.

[114] T.N. Tombaugh, N.J. McIntyre, The mini‐mental state examination: a comprehensive review, J Am Geriatr Soc 40(9) (1992) 922-935.

[115] C.L. Craig, A.L. Marshall, M. Sjöström, A.E. Bauman, M.L. Booth, B.E. Ainsworth, M. Pratt, U. Ekelund, A. Yngve, J.F. Sallis, International physical activity questionnaire: 12-country reliability and validity, Medicine & Science in Sports & Exercise 35(8) (2003) 1381-1395.

[116] W.J. Rejeski, S.L. Mihalko, Physical activity and quality of life in older adults, The Journals of Gerontology Series A: Biological sciences and medical sciences 56(suppl_2) (2001) 23-35.

[117] S. Elavsky, E. McAuley, R.W. Motl, J.F. Konopack, D.X. Marquez, L. Hu, G.J. Jerome, E. Diener, Physical activity enhances long-term quality of life in older adults: efficacy, esteem, and affective influences, Annals of Behavioral Medicine 30(2) (2005) 138-145.

[118] F. Langlois, T.T.M. Vu, K. Chassé, G. Dupuis, M.-J. Kergoat, L. Bherer, Benefits of physical exercise training on cognition and quality of life in frail older adults, The Journals of Gerontology: Series B 68(3) (2013) 400-404.

[119] J.G. Van Uffelen, M.J.C.A. Paw, M. Hopman-Rock, W. van Mechelen, The effect of walking and vitamin B supplementation on quality of life in community-dwelling adults with mild cognitive impairment: a randomized, controlled trial, Quality of Life Research 16(7) (2007) 1137-1146.

[120] E. Diener, R.A. Emmons, R.J. Larsen, S. Griffin, The satisfaction with life scale, Journal of personality assessment 49(1) (1985) 71-75.

[121] W. Pavot, E. Diener, Review of the satisfaction with life scale, Psychological assessment 5(2) (1993) 164.

[122] J.E. Ware, B. Gandek, Overview of the SF-36 health survey and the international quality of life assessment (IQOLA) project, Journal of clinical epidemiology 51(11) (1998) 903-912.

[123] R.D. Nebes, D.J. Buysse, E.M. Halligan, P.R. Houck, T.H. Monk, Self-reported sleep quality predicts poor cognitive performance in healthy older adults, The Journals of Gerontology: Series B 64(2) (2009) 180-187.

[124] T. Blackwell, K. Yaffe, S. Ancoli-Israel, J.L. Schneider, J.A. Cauley, T.A. Hillier, H.A. Fink, K.L. Stone, Poor sleep is associated with impaired cognitive function in older women: the study of osteoporotic fractures, The Journals of Gerontology Series A: Biological Sciences and Medical Sciences 61(4) (2006) 405-410.

[125] M. Cricco, E.M. Simonsick, D.J. Foley, The impact of insomnia on cognitive functioning in older adults, J Am Geriatr Soc 49(9) (2001) 1185-1189.

[126] D.J. Buysse, C.F. Reynolds, T.H. Monk, S.R. Berman, D.J. Kupfer, The Pittsburgh Sleep Quality Index: a new instrument for psychiatric practice and research, Psychiatry research 28(2) (1989) 193-213.

[127] J.C. Cole, S.J. Motivala, D.J. Buysse, M.N. Oxman, M.J. Levin, M.R. Irwin, Validation of a 3-factor scoring model for the Pittsburgh sleep quality index in older adults, Sleep 29(1) (2006) 112-116.

[128] A.P. Spira, S.A. Beaudreau, K.L. Stone, E.J. Kezirian, L.-Y. Lui, S. Redline, S. Ancoli-Israel, K. Ensrud, A. Stewart, Reliability and validity of the Pittsburgh Sleep Quality Index and the Epworth Sleepiness Scale in older men, Journals of Gerontology Series A: Biomedical Sciences and Medical Sciences 67(4) (2011) 433-439.

[129] J.S. Kuiper, M. Zuidersma, R.C.O. Voshaar, S.U. Zuidema, E.R. van den Heuvel, R.P. Stolk, N. Smidt, Social relationships and risk of dementia: a systematic review and meta-analysis of longitudinal cohort studies, Ageing research reviews 22 (2015) 39-57.

[130] S. Cohen, R. Mermelstein, T. Kamarck, H.M. Hoberman, Measuring the functional components of social support, Social support: Theory, research and applications, Springer1985, pp. 73-94.

[131] V.I. Kalapotharakos, M. Michalopoulos, S.P. Tokmakidis, G. Godolias, V. Gourgoulis, Effects of a heavy and a moderate resistance training on functional performance in older adults, Journal of strength and conditioning research 19(3) (2005) 652.
