# Supplementary material for: 12 weeks of strength training improves fluid cognition in older adults: A nonrandomized pilot trial
Source: PLoS One. 2021 Jul 22;16(7):e0255018. doi: 10.1371/journal.pone.0255018 (PMC8297768; doi:10.1371/journal.pone.0255018)
Supplement: Attachment — Submitted filename: renamed_c682b.docx [file pone.0255018.s007.docx]

**COMMENTS – Manuscript PONE-D-20-21856**

Title: “12 weeks of strength training improves fluid cognition in older adults: a nonrandomized pilot trial”

**Important note: This review pertains only to ‘statistical aspects’ of the study and so** **‘****clinical aspects’ [like medical importance,** **relevance of the study,** **‘clinical significance and** **implication(s)’ of the whole study, etc.] are to be evaluated [should be assessed] separately/independently. Further please note that any ‘statistical review’ is generally done under the assumption that (such) study specific methodological [as well as execution] issues are perfectly taken care of by the investigator(s). This review is not an exception to that and so does not cover clinical aspects {however, seldom comments are made only if those issues are intimately / scientifically related & intermingle with ‘statistical aspects’ of the study}. Agreed that ‘statistical methods’ are used as just tools here, however, they are vital part of methodology [and so should be given due importance].**

**COMMENTS:** Your ABSTRACT is well drafted but assay type. Please note that it is preferable [refer to item 1b of CONSORT checklist 2010: Structured summary of trial design, methods, results, and conclusions] to divide the ABSTRACT with small sections like ‘Objective(s)’, ‘Methods’, ‘Results’, ‘Conclusions’, etc. which is an accepted practice of most good/standard journals [including PLOS]. It will definitely be more informative then, I guess, whatever the article type may be.

Since it is a ‘proof of concept clinical trial’, sample size is not an issue. However, I am sure that the authors are aware of the well-known drawbacks of a single-arm design [a type of Quasi-experimental research], and it is often said that ‘alright to have ‘single-arm design’ (before-after study) when that is the only possibility’. It is very essential to keep those limitations in mind while interpreting results. Further, note that a classical/ideal clinical trial/study needs/requires a concurrently {but similarly} handled/treated appropriately selected/chosen control/comparison parallel group/arm.

Though measures/tools used are appropriate, most of them {may be even assessments tools used in this study} yield data that are in [at the most] ‘ordinal’ level of measurement [and not in ratio level of measurement for sure {as the score two times higher does not indicate presence of that parameter/phenomenon as double (for example, a Visual Analogue Scales VAS score or say ‘depression’ score)}]. Then application of suitable non-parametric test(s) is/are indicated/advisable [even if distribution may be ‘Gaussian’ (i.e. normal)].

When data are likely to be in ‘ordinal’ level of measurement, there is no sense in testing for ’normality’ (line 236: All data were considered continuous, examined for normality). Aany ’normality’ test [including Shapiro-Wilk test] cannot be powerful enough when sample size is this small. Moreover, I do not understand ‘why the differences between baseline and pre-intervention (lines 239-240 but later even differences from post-intervention) were evaluated using two-way mixed effects intraclass correlation coefficients (ICCs)’ instead of by direct comparison methods [like Wilcoxon’s Signed Ranked test – non-parametric equivalent/parallel of Paired t-test. {In my knowledge ‘differences’ are not evaluated using intraclass correlation coefficients (ICCs)} Though later (in lines 254-55) it is said that ‘Paired t-tests were used, comparing data from pre- and post-intervention’, however why ICCs’ are given in tables’? This is very confusing, in my opinion.

Titles & contents of Table 3 (Group means for cognitive outcomes at baseline, pre-intervention, and post-intervention) and Table 4 (Group means for physiological outcomes and questionnaire responses at baseline, pre-intervention, and post-intervention) are very confusing because they display mean and SD but in 4^th^ and 5^th^ column ICC and Effect Size (d) [where effect sizes are based on mean and the average standard deviation], are reported. They are in fact completely beyond my understanding [this may be very valid but definitely not according/adhering to current, known practice]. If it so, explain adequately.

Table 5 (Spearman’s rho correlations between pre- to post-intervention changes in fluid composite score and other outcome variables of interest) is similarly confusing. It is said in lines 263-4 that ‘Spearman’s rho values of 0.1, 0.3, and greater than 0.5 were classified as small, medium, and large associations, respectively’ and quoted reference [44] but I think this classification is for ‘effect size’ and not directly applicable for absolute values of ‘Spearman’s rho’. Is not that so? {please, again look at reference 44 to confirm}. Is what is said in lines 369-70 that “For the other outcome variables, pre-intervention values and raw pre- to post-intervention changes were used” correct? Readers should clearly know/understand regarding ‘What exactly is done?’. {Do you mean three items/values (Raw pre- to post-intervention changes in fluid composite score, pre-intervention values and raw pre- to post-intervention changes w.r.t. the other outcome variables) were/are used simultaneously?}

However, it should be noted that [despite ‘Limitations’ narrated in lines 496-509], this small study has many good points {use of appropriate tools, painstaking/good execution, etc., etc.} and has excellent ‘potential’ [only needs revision in light of above pointed out items], in my considered opinion.
