# Supplementary material for: 12 weeks of strength training improves fluid cognition in older adults: A nonrandomized pilot trial
Source: PLoS One. 2021 Jul 22;16(7):e0255018. doi: 10.1371/journal.pone.0255018 (PMC8297768; doi:10.1371/journal.pone.0255018)
Supplement: Attachment — Submitted filename: Response to Reviewers.docx [file pone.0255018.s008.docx]

Journal Requirements:

When submitting your revision, we need you to address these additional requirements.

1. Please ensure that your manuscript meets PLOS ONE's style requirements, including those for file naming. The PLOS ONE style templates can be found at

https://journals.plos.org/plosone/s/file?id=wjVg/PLOSOne_formatting_sample_main_body.pdf and

https://journals.plos.org/plosone/s/file?id=ba62/PLOSOne_formatting_sample_title_authors_affiliations.pdf

**We have checked the PLOS ONE style templates to ensure that our manuscript meets the style requirements, including those for file naming.**

2. Please include captions for your Supporting Information files at the end of your manuscript, and update any in-text citations to match accordingly. Please see our Supporting Information guidelines for more information: http://journals.plos.org/plosone/s/supporting-information.

**Captions for the supplementary material have been added to the end of the manuscript file. These include the two datasets provided in the first submission, and the new Figure S1 and Table S1 described below.**

[Note: HTML markup is below. Please do not edit.]

Reviewers' comments:

**We thank the Editor and all three Reviewers for the careful review of our manuscript and for their valuable comments. The suggested edits have improved our manuscript, and we hope that our revisions address their comments in a satisfactory manner. Our responses to their comments follow in bold format. We encourage the reviewers to please see the updated manuscript attached to view all revisions in context.**

Reviewer's Responses to Questions

1. Is the manuscript technically sound, and do the data support the conclusions?

The manuscript must describe a technically sound piece of scientific research with data that supports the conclusions. Experiments must have been conducted rigorously, with appropriate controls, replication, and sample sizes. The conclusions must be drawn appropriately based on the data presented.

Reviewer #1: Partly

Reviewer #2: Yes

Reviewer #3: Yes

2. Has the statistical analysis been performed appropriately and rigorously?

Reviewer #1: I Don't Know

Reviewer #2: Yes

Reviewer #3: Yes

3. Have the authors made all data underlying the findings in their manuscript fully available?

The PLOS Data policy requires authors to make all data underlying the findings described in their manuscript fully available without restriction, with rare exception (please refer to the Data Availability Statement in the manuscript PDF file). The data should be provided as part of the manuscript or its supporting information, or deposited to a public repository. For example, in addition to summary statistics, the data points behind means, medians and variance measures should be available. If there are restrictions on publicly sharing data—e.g. participant privacy or use of data from a third party—those must be specified.

Reviewer #1: Yes

Reviewer #2: Yes

Reviewer #3: Yes

4. Is the manuscript presented in an intelligible fashion and written in standard English?

PLOS ONE does not copyedit accepted manuscripts, so the language in submitted articles must be clear, correct, and unambiguous. Any typographical or grammatical errors should be corrected at revision, so please note any specific errors here.

Reviewer #1: Yes

Reviewer #2: Yes

Reviewer #3: Yes

5. Review Comments to the Author

Please use the space provided to explain your answers to the questions above. You may also include additional comments for the author, including concerns about dual publication, research ethics, or publication ethics. (Please upload your review as an attachment if it exceeds 20,000 characters)

Reviewer #1: Important note: This review pertains only to ‘statistical aspects’ of the study and so ‘clinical aspects’ [like medical importance, relevance of the study, ‘clinical significance and implication(s)’ of the whole study, etc.] are to be evaluated [should be assessed] separately/independently. Further please note that any ‘statistical review’ is generally done under the assumption that (such) study specific methodological [as well as execution] issues are perfectly taken care of by the investigator(s). This review is not an exception to that and so does not cover clinical aspects {however, seldom comments are made only if those issues are intimately / scientifically related & intermingle with ‘statistical aspects’ of the study}. Agreed that ‘statistical methods’ are used as just tools here, however, they are vital part of methodology [and so should be given due importance].

**Thank you for the clarification, the statistical review is well-received. We recognize the limitations of this study design and statistical approach and hope that our edits below help maximize their transparency. Despite these limitations, we consider it critical as a proof-of-concept study to report all the data to help investigators design and support future randomized controlled trials**

**Having a single-arm nonrandomized design allowed us to address practicality while maintaining a thorough proof-of-concept framework.** **Only changes in cognition were tested for significance because fluid cognition was the only outcome powered for *a priori* hypothesis testing. Thus, testing for significance in secondary outcomes was considered inappropriate (and would increase the likelihood of a Type I error). We instead opted to present the effect sizes in secondary outcomes.**

**Rest assured, the study was rigorously executed** **by qualified investigators using standard/well-established techniques. For example,** **participants had a 100% adherence rate, which is remarkably high for long-duration in-person training studies, and all operations were performed nominally (i.e. there were no severe adverse events or missed data collections). The findings, although preliminary, have high clinical significance and thus will be vital for advancing this line of research.**

**Similar language has been added to the Discussion section of the manuscript.**

COMMENTS: Your ABSTRACT is well drafted but assay type. Please note that it is preferable [refer to item 1b of CONSORT checklist 2010: Structured summary of trial design, methods, results, and conclusions] to divide the ABSTRACT with small sections like ‘Objective(s)’, ‘Methods’, ‘Results’, ‘Conclusions’, etc. which is an accepted practice of most good/standard journals [including PLOS]. It will definitely be more informative then, I guess, whatever the article type may be.

**Thank you, we agree that a structured abstract is more appropriate for this study and have added the suggested section titles.**

Since it is a ‘proof of concept clinical trial’, sample size is not an issue. However, I am sure that the authors are aware of the well-known drawbacks of a single-arm design [a type of Quasi-experimental research], and it is often said that ‘alright to have ‘single-arm design’ (before-after study) when that is the only possibility’. It is very essential to keep those limitations in mind while interpreting results. Further, note that a classical/ideal clinical trial/study needs/requires a concurrently {but similarly} handled/treated appropriately selected/chosen control/comparison parallel group/arm.

**Please note that sample size was computed in an *a priori* power analysis (see page 7, line 132) using a fluid cognition improvement effect size from a previous meta-analysis and accounting for anticipated attrition rates.**

**Yes, we are aware of the limitations of this single-arm design. We weighed our options during the proposal stage and decided that this was the preferred approach for feasibility and study funding. The primary purpose of this study was to demonstrate safety/feasibility and to generate preliminary data for use in future randomized controlled trial proposals (i.e., not to prove efficacy). We intentionally use language throughout such as “to determine whether a 12-week ST program could improve fluid cognition…” rather than “to determine if a 12-week ST program improves fluid cognition…”. We have made small edits throughout the manuscript for consistency.**

**Our cognition results simply support previous findings and suggest that they may extend to higher intensity training prescriptions. We agree with the importance of emphasizing that the limitations should be kept in mind while interpreting results. We clarify in the “Limitations” section that these are preliminary findings and that “a separate active control group is needed in future studies to accurately estimate the effects of training and establish causal relationships”. Similar language has been added throughout the manuscript to further clarify our intentions (e.g. “Further controlled studies are needed to confirm these findings.”).**

Though measures/tools used are appropriate, most of them {may be even assessments tools used in this study} yield data that are in [at the most] ‘ordinal’ level of measurement [and not in ratio level of measurement for sure {as the score two times higher does not indicate presence of that parameter/phenomenon as double (for example, a Visual Analogue Scales VAS score or say ‘depression’ score)}]. Then application of suitable non-parametric test(s) is/are indicated/advisable [even if distribution may be ‘Gaussian’ (i.e. normal)].

**We understand your point about ordinal variables. Many of the outcome measures in this study (and in this line of research) are ordinal variables (i.e. ordered categories). However, it is important to note that most have a wide range of values (e.g. 0 – 100) and have consistent and equally spaced intervals between their values (e.g. 90, 91, 92, 93…). Thus, it is standard to treat them like continuous data, so that’s what we chose to do for these analyses. For examples, please see many of the cognitive and psychological scales/indexes in references #6-16.**

When data are likely to be in ‘ordinal’ level of measurement, there is no sense in testing for ’normality’ (line 236: All data were considered continuous, examined for normality). Aany ’normality’ test [including Shapiro-Wilk test] cannot be powerful enough when sample size is this small. Moreover, I do not understand ‘why the differences between baseline and pre-intervention (lines 239-240 but later even differences from post-intervention) were evaluated using two-way mixed effects intraclass correlation coefficients (ICCs)’ instead of by direct comparison methods [like Wilcoxon’s Signed Ranked test – non-parametric equivalent/parallel of Paired t-test. {In my knowledge ‘differences’ are not evaluated using intraclass correlation coefficients (ICCs)} Though later (in lines 254-55) it is said that ‘Paired t-tests were used, comparing data from pre- and post-intervention’, however why ICCs’ are given in tables’? This is very confusing, in my opinion.

**As described above, all data were considered continuous, so testing for normality was appropriate. Out of the common formal tests of normality, Monte Carlo simulation results suggest that the Shapiro-Wilk test has the greatest power (Razali and Wah, 2011). Even if power was low given our sample size, we at least did our due diligence and used that information to determine how to compute/present descriptive statistics (means and standard deviations or medians and interquartile range) and effect sizes (adapted Cohen’s d or matched-pairs rank-biserial).**

**Razali NM, Wah YB. Power comparisons of shapiro-wilk, kolmogorov-smirnov, lilliefors and anderson-darling tests. Journal of statistical modeling and analytics. 2011 Jan 1;2(1):21-33.**

**You are correct that ICCs do not test for “differences”. Instead, they are measures/indexes of measurement reliability. Thus, ICCs are more appropriate than t-tests for baseline to pre-intervention comparisons because participants were asked to maintain their normal eating and exercise habits outside of the study, thus no changes in outcome measures were expected during the control period (from baseline to pre-intervention). Specifically, two-way mixed effects intraclass correlation coefficients (ICCs) with absolute agreement (ICC (3,1)) were selected to determine test-retest reliability (Koo and Li, 2015) because the same test operator (author TM) administered all of the tests. Note that these were only used for evaluating differences between baseline and pre-intervention (ICCs were not used for post-intervention data, we apologize for the confusion). We have edited the text from saying “Differences between baseline and pre-intervention…” to “Test-retest reliability over the control period (baseline and pre-intervention) was evaluated using…”. The rest of the manuscript is consistent with this language.**

**Koo TK, Li MY. A guideline of selecting and reporting intraclass correlation coefficients for reliability research. Journal of chiropractic medicine. 2016;15(2):155-63.**

**The lines you point out for paired t-tests are a separate paragraph and they describe tests performed only between pre- and post-intervention data (no baseline data). In addition, only changes in cognition were tested for significance because fluid cognition was the only outcome powered for *a priori* hypothesis testing.** **Therefore, these paired t-tests were used to determine the statistical significance of changes in cognition from pre- to post-intervention (presented with Figure 2).**

**We have considered breaking up the tables into multiple separate tables, but we find that to be more confusing since they deal with overlapping data (see below). We hope that by clarifying the purpose of each test, we have cleared up any confusion.**

| **Statistical Calculation** | **Data from the following time points are used:** | |
| --- | --- | --- |
| **ICC (3,1)** | **Baseline** | **Pre-intervention** |
| **Effect Sizes** | **Change from baseline to pre-** | **Change from pre- to post-** |
| **Paired T-test** | **Pre-intervention** | **Post-intervention** |

Titles & contents of Table 3 (Group means for cognitive outcomes at baseline, pre-intervention, and post-intervention) and Table 4 (Group means for physiological outcomes and questionnaire responses at baseline, pre-intervention, and post-intervention) are very confusing because they display mean and SD but in 4th and 5th column ICC and Effect Size (d) [where effect sizes are based on mean and the average standard deviation], are reported. They are in fact completely beyond my understanding [this may be very valid but definitely not according/adhering to current, known practice]. If it so, explain adequately.

**We hope the explanation above has cleared up any confusion. We believe that it’s best to keep the descriptive statistics together (mean ± SD, ICC, and effect size) in the same table. The table titles and reference to the tables now use the term “descriptive statistics”. Since this is a proof-of-concept study, we’d like to be as transparent as possible, providing different descriptions of the data to help inform future investigators. For example, without sharing these proof-of-concept findings, individual investigators are left to conduct their own internal pilot studies to support their proposals, and these initial pilot studies can require significant time and resources.**

Table 5 (Spearman’s rho correlations between pre- to post-intervention changes in fluid composite score and other outcome variables of interest) is similarly confusing. It is said in lines 263-4 that ‘Spearman’s rho values of 0.1, 0.3, and greater than 0.5 were classified as small, medium, and large associations, respectively’ and quoted reference [44] but I think this classification is for ‘effect size’ and not directly applicable for absolute values of ‘Spearman’s rho’. Is not that so? {please, again look at reference 44 to confirm}. Is what is said in lines 369-70 that “For the other outcome variables, pre-intervention values and raw pre- to post-intervention changes were used” correct? Readers should clearly know/understand regarding ‘What exactly is done?’. {Do you mean three items/values (Raw pre- to post-intervention changes in fluid composite score, pre-intervention values and raw pre- to post-intervention changes w.r.t. the other outcome variables) were/are used simultaneously?}

**Correlations are effect sizes because they are unit independent. They do not measure the actual variables, but the relationship between variables. We do confirm that the reference (Cohen 1992) uses those values: “For example, for the test that r = 0, small, medium, and large ESs are, respectively, the population rs 0.1, 0.3, and 0.5”. Similar to above, we are just trying to be transparent and present all the data needed (but usually difficult to obtain) to do *a priori* analyses for future studies.**

**Cohen J. Statistical power analysis. Current directions in psychological science. 1992;1(3):98-101.**

**Thank you for catching this miscommunication (“For the other outcome variables…”). Only raw changes from pre- to post-intervention were used. So all Spearman’s rho correlation coefficients listed describe the relationship between pre- to post-intervention changes in fluid cognition and pre- to post-intervention changes in other measures listed. We have made minor corrections in the methods, results/table, and discussion to help clarify.**

However, it should be noted that [despite ‘Limitations’ narrated in lines 496-509], this small study has many good points {use of appropriate tools, painstaking/good execution, etc., etc.} and has excellent ‘potential’ [only needs revision in light of above pointed out items], in my considered opinion.

**Thanks again for the detailed review and for equally weighing the strengths and limitations of this study. We believe that the revisions above will help readers interpret and use the results of this study appropriately. Most importantly, we provide simple descriptive statistics and all the raw data for investigators to use in their own proposals’ *a priori* analyses.**

Reviewer #2: Dear authors,

Congratulations for the work done. The paper is wonderfully written, and all analysis performed are perfectly convenient to answer the scientific questions raised. In addition, I find the research topic of a crucial interest for the proper development of the resistance training and health research field. Therefore, I proposed highlighting the manuscript at the PLOS ONE homepage.

**Thank you so much for the kind words. We agree that there is widespread interest in the field for the potential applications of this (and similar) work. We hope that the information provided in this paper helps accelerate the optimization of training prescription methods for the rapidly increasing population of older adults.**

Reviewer #3: In this manuscript, Macaulay et al. investigate whether strength training can improve fluid cognition in healthy older adults. It is well-written and does a great job of highlighting existing literature in the context of the current study. Their overall conclusions that a 12-week strength training program improves fluid cognition but not crystallised cognition are well-supported by their results and have far-reaching clinical implications. Overall, I found this to be an interesting study. Please find below some suggestions to improve the quality of the manuscript.

**Thank you for the helpful suggestions. We are glad that you enjoyed the paper.**

Major Comments:

- 70% of participants in this study are female. Sex differences have been reported in age-related cognitive decline (1) and in exercise-related changes in cognition (2). While this exploratory analysis may not be powered for explicit hypothesis testing related to sex differences, it would be interesting to see sex-specific results reported for this study. Authors could perform this by creating an additional (supplemental) figure replicating Figure 2 and demonstrating the trends for crystallised and fluid composites and individual tasks scores separately for males and females, and briefly discussing any observed differences in the discussion.

1. McCarrey, A. C., An, Y., Kitner-Triolo, M. H., Ferrucci, L., & Resnick, S. M. (2016). Sex differences in cognitive trajectories in clinically normal older adults. Psychology and aging, 31(2), 166.

2. Barha, C. K., Davis, J. C., Falck, R. S., Nagamatsu, L. S., & Liu-Ambrose, T. (2017). Sex differences in exercise efficacy to improve cognition: a systematic review and meta-analysis of randomized controlled trials in older humans. Frontiers in neuroendocrinology, 46, 71-85.

**We did perform these sex-comparison calculations for the same reasons you mentioned, and then decided not to include them in the manuscript due to length. But we like your idea of including them as a supplemental figure. Since we are primarily interested in the changes in fluid cognition composite score (and there were no changes in crystallized cognition) we are choosing to only include that measure. Furthermore, with such small sample sizes (females, n=14; males, n=6), adding the five individual instrument scores gets too far into the weeds, so we are not including them. We present these in the figure as individual participant data points so that readers can better visualize the variability in pre- to post-intervention changes (Figure S1). We note in the discussion that these findings should be interpreted with caution due to the small (and uneven) sample sizes.**

**We have a similar comparison based on age that we are including in Figure S1. Again, these findings should be interpreted with caution due to the small sample sizes.**

**Figure S1.** **Individual changes in fluid composite score from pre- to post-intervention based on A) sex and B) age. A) Fluid composite scores increased from pre- to post-intervention in both females (+7.4 ± 6.2 Standard Units) and males (+8.5 ± 3.1 Standard Units). B) Using the median age at baseline (68.3 years), we compared the 10 participants below the median age (64.2 ± 2.5 years) and the 10 participants above the median age (74.0 ± 3.7 years). Fluid composite scores increased from pre- to post-intervention in both the participants below the median age (+8.7 ± 5.8 Standard Units) and the participants above the median age (+6.7 ± 4.9 Standard Units).**

- Authors report that participants did not appear to change their exercise habits outside of the study (Page 16, lines 321-322). However, it would be interesting to know whether participants with a higher baseline exercise activity outside of the study had stronger improvements in their fluid cognition.

**We have added a supplementary table (Table S1) for Spearman correlations that were computed between raw fluid composite score changes and pre-intervention values in other outcome variables:**

**Table S1:** **Spearman’s rho correlations between pre- to post-intervention changes in fluid composite score and pre-intervention values of outcome variables of interest (N = 20)**

| **Measure** | **Spearman’s rho** |
| --- | --- |
| **Age** | **-0.26** |
| **IPAQ** | **-0.20** |
| **Body Fat Percentage** | **-0.03** |
| **Lean Body Mass** | **-0.01** |
| **Fat Mass** | **0.11** |
| **Systolic Blood Pressure** | **-0.33** |
| **Diastolic Blood Pressure** | **-0.08** |
| **Heart Rate** | **-0.05** |
| **Total Body Strength** | **0.18** |
| - **Lower Body** | **0.17** |
| - **Upper Body** | **0.22** |
| **Margaria Power** | **0.22** |
| **Timed Up and Go** | **0.11** |
| **Y-Balance^#^** | **0.36** |
| **Habitual Gait Speed** | **0.14** |
| **PSQI Score** | **-0.22** |
| **ISEL Score** | **0.15** |

**These correlation analyses were used to explore factors that may influence fluid cognitive enhancement after RT. Raw pre- to post-intervention changes in fluid cognition composite score were compared to the single time point of pre-intervention values. For example, the negative spearman’s rho for IPAQ suggests that lower IPAQ responses (lower physical activity levels) at pre-intervention were associated with greater improvements in fluid cognition composite score from pre- to post-intervention.**

**IPAQ = International Physical Activity Questionnaire.**

**PSQI = Pittsburgh Sleep Quality Index. Lower scores indicate better sleep quality, scores ≥ 5 are classified as clinically poor sleep quality.**

**ISEL = International Support Evaluation List. Higher scores indicate greater social support, up to a maximum of 16.**

**# Four participants were not able to perform all six reach directions at baseline and/or pre-intervention, so their data were excluded from analyses (N = 16).**

- Authors note that participants were allowed to progress loads at their own pace (Page 8, lines 156-157), but do not investigate whether this load progression is correlated with change in fluid cognition. Correlating the load progression with changes in fluid cognition would provide insight into a potential mechanism underlying the exercise-related improvements in fluid cognition. This may also provide more insight into the unexpected negative correlations observed between changes in muscular strength and changes in fluid cognition.

**Unfortunately, this data is no longer available; however, we would like to suggest that the correlation between load progression and changes in fluid cognition would be similar to the correlation between changes in muscular strength and changes in fluid cognition. The reason is that “supervised autoregulation allowed participants to progress loads at their own pace based on maximum performance on the last set of each exercise”. In other words, the loads used in the first 2-3 sets were based on participants’ performance in the previous session and feedback from the participant (i.e. “I’m a bit tired today” or “I’m feeling great today”), and for the last set participants were told to do the same number of reps but “if you can do one or two more, please do more”. We did not find confirmation in the literature for this assumption that load progression rate and pre- to post-intervention strength changes are highly correlated. But from anecdotal experience, the loads used in the last training session (Week 12, 3 sets, 4 reps) were only slightly lower than the loads used in 4-6 repetition maximum (RM) testing post-intervention.**

**As mentioned in the discussion, we also think that it’s important for future studies to further investigate the correlation between changes in fluid cognition and changes in strength as a potential mechanism underlying exercise-induced cognitive enhancement.**

Minor Comments:

- In table 5, IPAQ is defined in the caption, but correlation for IPAQ itself doesn’t seem to be included in the table.

**Thank you for catching that. We removed IPAQ responses from the tables since these were just used to confirm that subjects followed the instructions to not change their physical activity habits outside of the study. We deleted the line you mentioned.**

- In table 5, it might be helpful to clarify what the directionality of the correlations indicate in the caption.

**Rather than clarifying for each measure, we provide example interpretations for Spearman’s rho in the Table 5 caption.**

**“****Note that these correlations should be understood in the context of changes. For example, the small positive correlation with Margaria power suggest that greater increases in fluid composite score are associated with greater increases in Margaria power. However, the small negative correlation with total body strength suggest that greater increases in fluid composite score are associated with smaller increases in total body strength.”**

- On Page 19, lines 400-402, authors say that fluid improvement observed is equivalent to 14.7 years of mean population fluid decline, but earlier in the paragraph, they state that yearly decline is -0.104 points/year. Based on the yearly decline, wouldn’t an increase of 1.53 points (0.104*14.7) be equivalent o 14.7 years of decline?

**Thank you for calling our attention to this. The decline of -0.104 points/year used different scaled scores (population mean of 10 and standard deviation of 3) than the normalized scores we used in this study (population mean of 100 and standard deviation of 15), even though the measures were the same. Thus, while our math is correct, we can improve the communication by presenting the changes in terms of population standard deviations rather than by “points”. The section now reads:**

**“After adjusting for gender and education, age has a significant -0.0347 SD/year effect on fluid composite score (using population SD). In our study, the mean uncorrected fluid composite score improved by 7.7 standard units, or 0.51 SD, from pre- to post-intervention. Using the regression coefficient above, we calculate that the fluid cognition improvement observed in the current study is equivalent to 14.7 years of mean population fluid cognition decline.”**

- The resolution of Figure 2 makes it difficult to read properly.

**We have fixed the resolution issue. Thank you!**

6. PLOS authors have the option to publish the peer review history of their article (what does this mean?). If published, this will include your full peer review and any attached files.

If you choose “no”, your identity will remain anonymous but your review may still be made public.

Do you want your identity to be public for this peer review? For information about this choice, including consent withdrawal, please see our Privacy Policy.

Reviewer #1: No

Reviewer #2: No

Reviewer #3: Yes: Elvisha Dhamala

While revising your submission, please upload your figure files to the Preflight Analysis and Conversion Engine (PACE) digital diagnostic tool, https://pacev2.apexcovantage.com/. PACE helps ensure that figures meet PLOS requirements. To use PACE, you must first register as a user. Registration is free. Then, login and navigate to the UPLOAD tab, where you will find detailed instructions on how to use the tool. If you encounter any issues or have any questions when using PACE, please email PLOS at figures@plos.org. Please note that Supporting Information files do not need this step.

**We uploaded figure files to PACE and downloaded the PACE-adjusted files. These PACE-adjusted files are attached to this response submission to PLOS.**
